# Ecosystem services provided by bromeliad plants: A systematic review

**DOI:** 10.1002/ece3.5296

**Published:** 2019-05-29

**Authors:** Geraldine Ladino, Fabiola Ospina‐Bautista, Jaime Estévez Varón, Lucie Jerabkova, Pavel Kratina

**Affiliations:** ^1^ Departamento de Ciencias Biológicas Universidad de Caldas Manizales Colombia; ^2^ Department of Geography King's College London London UK; ^3^ School of Biological and Chemical Sciences Queen Mary University of London London UK

**Keywords:** biodiversity, bromeliad plants, climate regulation, disease, ecosystem services, microecosystems, neotropics, pharmaceutical potential, water storage

## Abstract

The unprecedented loss of biological diversity has negative impacts on ecosystems and the associated benefits which they provide to humans. Bromeliads have high diversity throughout the Neotropics, but they have been negatively affected by habitat loss and fragmentation, climate change, invasive species, and commercialization for ornamental purpose. These plants provide direct benefits to the human society, and they also form microecosystems in which accumulated water and nutrients support the communities of aquatic and terrestrial species, thus maintaining local diversity. We performed a systematic review of the contribution of bromeliads to ecosystem services across their native geographical distribution. We showed that bromeliads provide a range of ecosystem services such as maintenance of biodiversity, community structure, nutrient cycling, and the provisioning of food and water. Moreover, bromeliads can regulate the spread of diseases, and water and carbon cycling, and they have the potential to become important sources of chemical and pharmaceutical products. The majority of this research was performed in Brazil, but future research from other Neotropical countries with a high diversity of bromeliads would fill the current knowledge gaps and increase the generality of these findings. This systematic review identified that future research should focus on provisioning, regulating, and cultural services that have been currently overlooked. This would enhance our understanding of how bromeliad diversity contributes to human welfare, and the negative consequences that loss of bromeliad plants can have on communities of other species and the healthy functioning of the entire ecosystems.

## 1 INTRODUCTION

Diversity across all levels of biological organization is vital to a healthy ecosystem functioning (Naeem, Duffy, & Zavaleta, 2012; Tilman, Isbell, & Cowles, 2014) and to a range of services that ecosystems provide to the society (Cardinale et al., 2012; Gamfeldt et al., 2013; Millennium Ecosystem Assessment, 2005). Therefore, the ongoing loss of biodiversity and the changes to species interactions can negatively impact ecosystem services, which support human needs and the safeguarding of their well‐being (Balvanera et al., 2014; Isbell, Tilman, Polasky, & Loreau, 2015). Some species can provide habitats for the entire ecological communities and deliver services that may have been previously overlooked. Thus, it is essential to understand the role of these species in the ecosystems and to ensure stable provisioning of ecosystem services (Hooper et al., 2005).

The Bromeliaceae family includes 3,403 species of vascular plants that are widely distributed across the Neotropics (Ulloa et al., 2017). Bromeliads are slow‐growing and long‐lived plants (Benzing, 1990; Schmidt & Zotz, 2000) that become fertile between the 9th and 18th year of their life, depending on the species. For instance, *Tillandsia pauciflora* requires 8–10 years to flower (Benzing, 1990), *Tillandsia deppeana* requires 11 years to flower, *Catopsis sessiliflora* and *C. nutans* require 9 years to flower, whereas *T. multicaulis* and *T. punctulata* flower for the first time after 13 and 18 years, respectively (Hietz, Ausserer, & Schindler, 2002). Bromeliads are distributed from the south of the United States to the southeast of South America and one species is native to Western Africa (Benzing, 1990). They occur from deserts to rainforests, and from 51 m above sea level to high‐altitude mountains more than 4,000 m above sea level (Smith & Till, 1998). However, these plants are the most abundant and diverse in habitats with high precipitation and humidity and also at mid‐elevations (Gentry & Dodson, 1987; Krömer, Kessler, Gradstein, & Aceby, 2005). Previous works have focused on the diversity of bromeliads in ecosystems such as mesophyllous forests, urban areas, and plantations, and their contribution to nitrogen, carbon, and water cycling (Griffiths, 1988; Haro‐Carión, Lozada, Navarrete, & Konning, 2009; Koster, Kreft, Nieder, & Barthlott, 2013; Ngai & Srivastava, 2006; Reich, Ewel, Nadkarni, Dawson, & Evans, 2003). However, there is currently no study that systematically evaluates the role of these plants in providing essential ecosystem functions and services.

The epiphytic life strategy and the formation of water tanks are some of the key evolutionary innovations that facilitated the success of many bromeliad species (Benzing, 2000; Givnish et al., 2011; McWilliams, 1974; Smith, 1989). Epiphytic bromeliads are taxonomically diverse, and they surpass other families in terms of biomass and also dominate the epiphytic vascular flora of Neotropical forests (Benzing, 1990). The leaves of many bromeliad species overlap at the base and form tanks where the plants store rainwater (Zotz & Vera, 1999). There are 24 genera of tank bromeliads, including the subfamilies Tillandsioideae, Bromelioideae, Pitcairnioideae, Brocchioideae, and Lindmanioideae (Males & Griffiths, 2017). Tank formation and epiphytism entail that these bromeliad species do not depend on their substrate for water and nutrient uptake, and it allows them to survive in adverse environmental conditions (Benzing, 1990; Schulte, Barfuss, & Zizka, 2009; Silvestro, Zizka, & Schulte, 2014). Moreover, the ability to accumulate water and nutrients allows both wild and ornamental bromeliads to form aquatic microecosystems, harboring diverse assemblages of invertebrate and vertebrate species (Greeney, 2001; Killick, Blanchon, & Large, 2014). Bromeliads, thus, substantially contribute to the maintenance of biodiversity and ecological interactions that underlie ecosystem function and services (Lopez, Rodrigues, & Rios, 1999; Richardson, 1999).

The IUCN Red List of Threatened Species (IUCN, 2017) includes 146 bromeliad species, of which 13 species are critically endangered (Appendix S1). The main causes of the decline in bromeliad populations and species loss are degradation and loss of forest habitats (Siqueira Filho & Tabarelli, 2006), climate change (Wagner & Zotz, 2018; Zotz, Bogusch, Hietz, & Ketteler, 2010), and invasive species, such as the invasive weevil *Metamasius callizona* that has devastated native bromeliad populations in Florida, United States (Cooper, Frank, & Cave, 2014). The loss of bromeliads and associated invertebrate and vertebrate communities could negatively affect the surrounding ecosystems (Dézerald et al., 2018; Looby & Eaton, 2014) and compromise services provided by the bromeliads and the associated animals. Although many studies have focused on aquatic communities inhabiting bromeliads, the contributions that these plants provide to ecosystem services remain poorly understood. Therefore, we assess the overall contribution of the Bromeliaceae family to ecosystem services through a systematic review of published studies. We aimed to compare the level of understanding among the four main categories of ecosystem services (see Methods section) and to identify those services that have been overlooked in the current literature. We also compared the state of knowledge in different parts of Neotropics and identified those countries where future research efforts should increase. This study highlights the role of bromeliads as providers of numerous ecosystem services through their diverse characteristics and traits.

## 2 METHODS

Humans always have a close relationship with the ecosystems in which they live and from which they obtain numerous benefits. These benefits, known as ecosystem services, are classified into four categories: provisioning services: services that contribute to the satisfaction of material needs such as food or drinking water; regulating services: services that include processes such as climate, disease, or water regulation; supporting services, which are processes that enable the provision of the other services; and cultural services: services that contribute to recreational, aesthetic, spiritual, and cultural heritage (Millennium Ecosystem Assessment, 2005). Although some of the classification that categorize ecosystem services only recognize three of these categories (Haines‐Young & Potschi, 2018), and treat supporting ecosystem services as ecosystem functions (e.g., nutrient cycling, primary production), in this paper, we referred to these processes as supporting ecosystem services, as recognized by Iverson, Echeverria, Nahuelhual, and Luque (2014) and used by Mortimer, Saj, and David (2018), and Wrede, Beermann, Dannheim, Gutow, and Brey (2018).

We performed a systematic review of ecosystem services provided by bromeliad plants following the PRISMA (Preferred Reporting Items for Systematic Reviews and Meta‐Analyses) methodology, and an evidenced‐based strategic search was carried out using the Scopus database. PRISMA is a protocol that provides all necessary steps to reach more objective and reproducible systematic reviews, with the goal to increase the transparency and reproducibility of science.

We searched primary research studies and reviewed articles published between January 1981 and June 2017, because the term “ecosystem services” was used for the first time in 1981 (Ehrlich & Ehrlich, 1981). We used the wildcard (*), which allows and includes all the keywords that start with the preceding characters. We included the following search terms: “bromelia*” AND “ecosystem service*”, OR “ecosystem good*” OR “environmental service*” OR “environmental good*” OR “environmental benefit*” OR “ecological service*” OR “ecological good*” OR “ecological benefit*” OR “regulati*” OR “climate regulati*” OR “weather” OR “disease regulati*” OR “disease*” OR “water regulati*” OR “water purificati*” OR “water” OR “pollinati*” OR “provision*” OR “resource*” OR “potable water*” OR “food*” OR “genetic resource*” OR “support*” OR “supply*” OR “sustenance” OR “primary produc*” OR “nutri*” OR “nutrient* cycl*” OR “cultural*” OR “spiritual” OR “religion*” OR “recreation*” OR “esthetic*” OR “inspiration*” OR “cultural heritage.”

We used studies that reported contributions to ecosystem services provided by bromeliads (a) as a microecosystem that forms a habitat for microorganisms, aquatic invertebrates, and some vertebrate species or (b) as organisms themselves. We extracted the following information from the papers: (a) title, (b) year of publication, (c) author list, (d) keywords of the article, (e) study area, (f) type of ecosystem services being analyzed (supporting services, provisioning services, regulating services, or cultural services), together with the meaning of each ecosystem services, category of Millennium Ecosystem Assessment (2005), (g) type of contribution to the service, that is, if it is generated by an organism that is part of the ecosystem or by a microecosystem, (h) specific ecosystem service provided by bromeliads (food, water, disease regulation, etc.), and (i) the quantitative estimate of the contribution of bromeliads to the ecosystem services. Although the provisioning of ecosystem services by bromeliads would likely differ among different species and biogeographical regions, there were not enough published studies to systematically evaluate this hypothesis.

## 3 RESULTS

**Figure 1:**
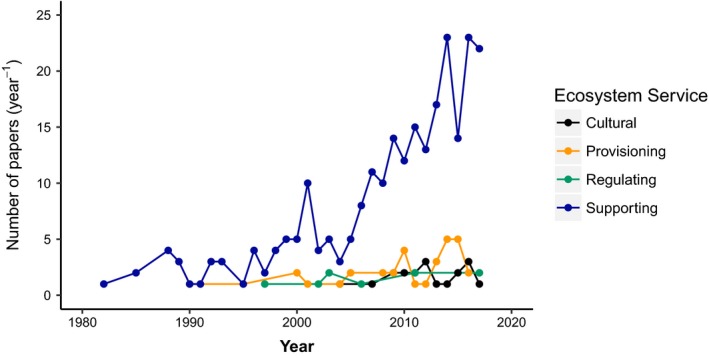
The number of peer‐reviewed publications in Scopus database that investigated ecosystem services provided by bromeliad plants between 1981 and 2017 has increased substantially for supporting services, but it has remained understudied for the three other types of ecosystem services. A total of 311 papers were systematically evaluated

**Figure 2:**
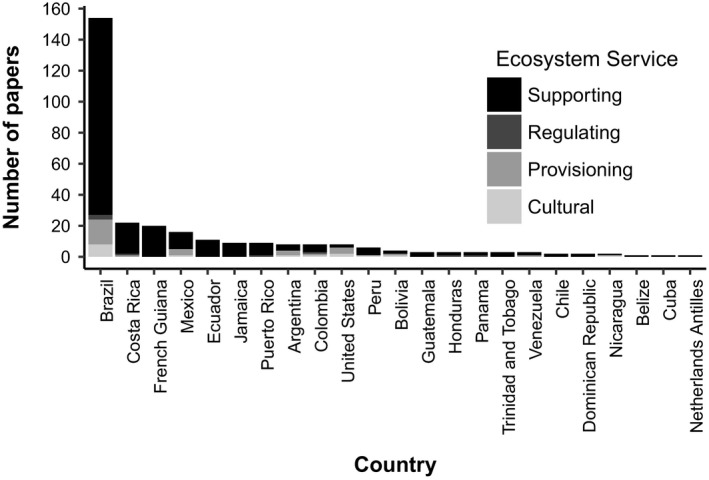
Total number of peer‐reviewed studies of cultural, provisioning, regulating, and supporting services provided by bromeliad plants in each Neotropical country (Search in Scopus database between 1981 and 2017)

**Figure 3:**
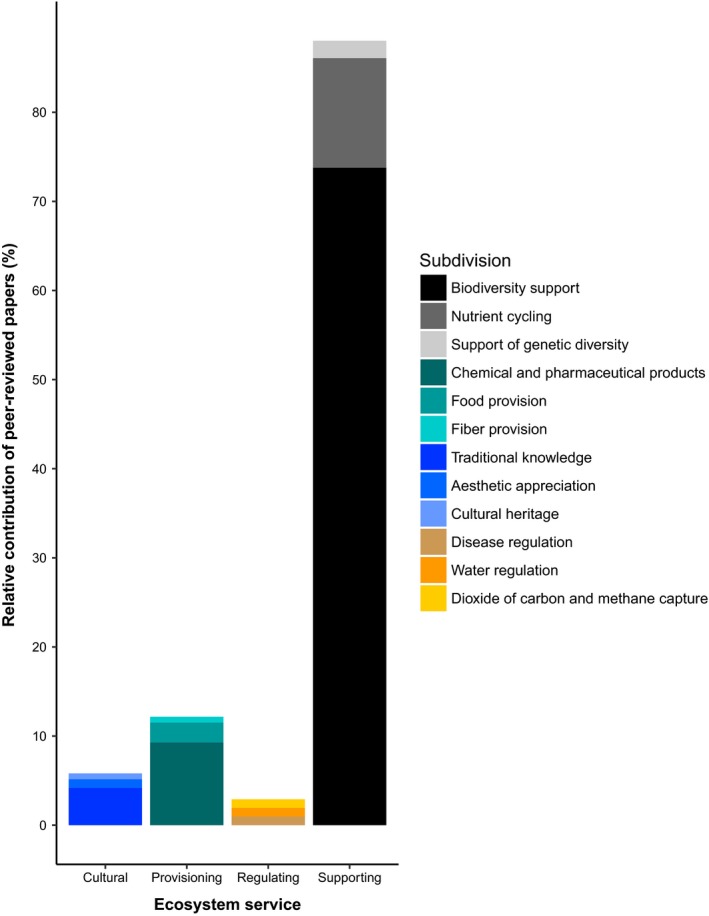
Relative contribution of peer‐reviewed papers that investigated the four main categories of ecosystem services provided by bromeliads. Different colors indicate specific types (subdivision) of each of the four main categories

We identified 985 articles of which 311 met the criteria of reporting the bromeliad species and the associated ecosystem services. There was a strong increase from 1980 to 2017 in the number of publications reporting the contribution of the Bromeliaceae family to ecosystem services (Figure 1). This increase in research was mostly driven by studies about the supporting services provided by bromeliads (Figure 1). Majority of these studies were conducted in Brazil, Costa Rica, and French Guiana (Figure 2). Bromeliads provide ecosystem services through (a) serving as microecosystems for aquatic organisms in 67.2% cases and (b) directly as plant species in 32.8% cases (Figure 3). The biodiversity support services through habitat, resources, shelter, and a source of freshwater were identified as the most important services and a focus of the majority of the studies.

### 3.1 Supporting services

A total of 88.02% of papers reported supporting services provided by bromeliads, 81% presented maintenance of biodiversity as supporting various ecosystem processes. Seventeen percent of studies focused on nutrient cycling and 2% studied genetic diversity (Figure 3). These studies were performed in 23 countries, but the majority of the studies were conducted in Brazil (Figure 2).

#### 3.1.1 Biodiversity support

A total of 117 studies (47.36%) reported tank bromeliads as a habitat for aquatic communities, composed of bacteria, plants, fungi, invertebrates, and vertebrates (Carrias, Cussac, & Corbara, 2001; Frank & Lounibos, 2009; Montero, Feruglio, & Barberis, 2010). The papers that investigated how aquatic taxa inhabit and utilize bromeliads are presented in Appendix S2A. Forty‐six out of 117 studies reported the effects of tank bromeliads on aquatic community structure (Jabiol, Corbara, Dejean, & Céréghino, 2009; Marino, Srivastava, & Farjalla, 2013; Richardson, Rogers, & Richardson, 2000; Wittman, 2000) and biotic interactions (Canela & Sazima, 2003; Céréghino, Leroy, Dejean, & Corbara, 2010). In addition to biotic factors, tank bromeliads can influence community structure via their size, number of leaves, detritus content, and the volume of water they hold (Armbruster, 2002; Cardoso, Lourenço‐de‐Oliveira, Codeço, & Motta, 2015; Carrias et al., 2014; González, Romero, & Srivastava, 2014; Kratina, Petermann, Marino, MacDonald, & Srivastava, 2017; Petermann, Farjalla, et al., 2015; Petermann, Kratina, Marino, MacDonald, & Srivastava, 2015; Srivastava, 2006; Talaga et al., 2017). Finally, intraspecific genetic variation of bromeliads influences the structure of the communities that inhabit them, mainly through changes in species richness, abundance, and trophic structure (Zytynska, Khudr, Harris, & Preziosi, 2012).

Bromeliads facilitate the growth of other plants and microorganisms by serving as nurse plants (Barberis, Boccanelli, & Alzugaray, 2011; Looby, Hauge, Barry, & Eaton, 2012; Tsuda & Castellani, 2016). For instance, coastal sand dunes receive nutrients and organic matter accumulated by the bromeliad *Vriesea friburgensis*, favoring the establishment of other plant species such as *Eupatorium casarettoi *and *Tibouchina urvillean* (Tsuda & Castellani, 2016). Moreover, water tanks of bromeliads are ideal habitats for seed germination of some species, such as *Clusia hilariana* (Tsuda & Castellani, 2016). The fungicidal activity of some bromeliads can also influence the surrounding microbial community. For instance, tank bromeliad *Bromelia pinguin* hosts basidiomycetes, which alter soil nutrient cycles and diversity of microbial and fungal communities (Looby & Eaton, 2014; Looby et al., 2012).

Bromeliads contribute to the genetic diversity of animals and plants they host by facilitating their allopatric speciation. Habitats formed by tank bromeliads have been shown to favor diversification and endemism of some groups, such as ostracods of genus *Elpidium*, carabid beetles of genus *Platynus* (Liebherr, 2005; Little & Hebert, 1996) and *Copelatus* and *Aglymbus* genera of diving beetles (Copelatinae) (Balke et al., 2008).

#### 3.1.2 Nutrient cycling

Tank bromeliads facilitate availability and redistribution of nutrients through the aquatic microecosystems they form, in particular, through the litter decomposition in the tank (Appendix S2B). Potassium, P, N, Ca, Mg, Fe, and Al from leaf litter, associated organisms, and accumulated rainwater are available for species living inside the bromeliads. In addition, carnivorous aquatic plants associated with bromeliads, such as *Utricularia cornigera* and *Utricularia nelumbifolia,* provide organic matter to the bromeliad microecosystem (Płachno, Stpiczyńska, Davies, Świątek, & Miranda, 2017). Beyond redistributing nutrients within the aquatic ecosystems, bromeliads can modify their substrates through the transformation of nutrients (Pett‐Ridge & Silver, 2002). For instance, *Vriesea bituminosa* produces a sticky exudate in which a high diversity of insects is trapped, contributing to the nutrient cycle (Monteiro & Macedo, 2014).

Most of the organisms inhabiting tank bromeliads are essential for nutrient cycling. Ants *Camponotus femoratus* and *Pachycondyla goeldii* engage in mutualistic associations called myrmecotrophy that provides nitrogen for the bromeliad *Aechmea mertensii* through the root of the plant. The presence of ant gardens in bromeliad roots mass favors the vegetative and reproductive traits that enhance bromeliad fitness (Leroy et al., 2013, 2011; Leroy, Corbara, Dejean, & Céréghino, 2009a, 2009b). Spider communities bring nitrogen to *Bromelia balansae*, *Ananas comosus*, and *Achmea distichantha* from surrounding forest ecosystems (Gonçalves, Mercier, Mazzafera, & Romero, 2011). The carbon and nitrogen cycles associated with bromeliads can be strongly influenced by the presence of damselflies and their interactions with other organisms (Atwood et al., 2013; Atwood, Hammill, Srivastava, & Richardson, 2014; Ngai & Srivastava, 2006). Vertebrates also contribute to the nutrient cycling; feces of tree frogs can bring an average of 27.7% of the total nitrogen into the bromeliad *Vriesea bituminosa* (Romero et al., 2010).

Ecological communities inhabiting tank bromeliads are mostly fueled by nutrients derived from detritus of decomposed leaves (Ngai & Srivastava, 2006; Romero, Mazzafera, Vasconcellos‐Neto, & Trivelin, 2006). However, primary productivity of unicellular algae and cyanobacteria become more important in the ecosystems with low canopy cover and high light availability (Brouard et al., 2012; Carrias et al., 2001; Haubrich, Pires, Esteves, & Farjalla, 2009; Klann, McHenry, Montelongo, & Goffredi, 2016; Kotowska & Werner, 2013; Marino, Guariento, Dib, Azevedo, & Farjalla, 2011). Nitrogen from microorganisms and their interactions with other taxa provides an additional source of nutrition to bromeliads and their communities (Inselsbacher et al., 2007). For example, feces of the spider *Psecas chapoda* associated with assemblages of mineralizing bacteria increases the absorption of nitrogen by *Bromelia balansae* (Gonçalves, Hoffmann, Mercier, Mazzafera, & Romero, 2014).

### 3.2 Provisioning services

Of the 12.17% of the studies describing provisioning services, 9.29% focused on chemical and pharmaceutical products, 2.24% on food, and 0.64% on fiber (Figure 2).

#### 3.2.1 Chemical and pharmaceutical products

The chemical products derived from tank‐less and tank bromeliads include enzymes and secondary metabolites used for medicinal purposes in the treatment of respiratory diseases, diabetes, inflammation, and gastrointestinal disorders (Hilo de Souza et al., 2016). For example, *Ananas comosus* and *Bromelia* sp. have anti‐inflammatory, analgesic, anti‐infective, and homeostatic effects (Darshan & Doreswamy, 2004). Moreover, the extracts from different bromeliad species have antibacterial activity (da Silva, Franco, Damasceno, Silva Almeida, & Costa, 2014; Fernandes, Aquino, Gouveia, Almeida, & Costa, 2015; Appendix S3A). Hornung‐Leoni (2011) studied the medicinal properties of 20 bromeliad species from 13 Latin American countries and found that several bromeliads have medicinal properties with good potential for drug synthesis (Appendix S3B).

#### 3.2.2 Food provision

The only bromeliad that has been commercially cultivated, consumed, and marketed worldwide is *Ananas comosus* due to its flavor and nutritional value (Riya et al., 2014). However, other species have high potentials for use in the food industry (Nunes, Lucena, Santos, & Albuquerque, 2015) and could be used as alternative food sources during drought periods (Juvik et al., 2017). In 15 Latin American countries, 24 species of the Bromeliaceae family are a source of food (Hornung‐Leoni, 2011). The use of bromeliads as food due to their nutritional content has been documented in *Bromelia laciniosa*, which is rich in carbohydrates and is a good source of flavonoids (Chaves, Silva, Alessandro, Albuquerque, & Barros, 2015). Moreover, *Bromelia karatas* has antioxidant activity (Osorio et al., 2017), fruits of *Bromelia antiacantha*, contain 45% carbohydrates, 18% lipids, 30% palmitic acid, 30% linoleic acid, and 20% oleic acid (Santos, Freitas, Deschamps, & Biavatti, 2008), and *Hechtia montana* is consumed in Sonora, Mexico (Feiger & Yetman, 2000; Appendix S3B).

#### 3.2.3 Fiber provision

Bromeliaceae are economically used for the production of fibers (Acebey, Krömer, Maass, & Kessler, 2010), with 19 bromeliad species used as a fiber source in seven Latin American countries (Hornung‐Leoni, 2011). For example, *Ananas* in Venezuela (Leal & Amaya, 1991) and *Bromelia pinguin* in Mexico (Pío‐León et al., 2009) are used as sources of fiber for production of clothing, strings, rope, fishing lines, and nets.

### 3.3 Regulating services

Of the 3.52% of the studies describing regulating services, 1.60% focused on disease regulation, 0.96% on water regulation, and 0.96% on carbon dioxide and methane capture (Figure 2; Appendix S4A).

#### 3.3.1 Disease regulation

Tank bromeliads form habitats for some species of mosquitoes that are disease vectors. These mosquito species include *Aedes aegypti*, *A. albopictus*, *Haemagogus *sp., and *Culex *sp., which are vectors of dengue, yellow fever, zicka, chikungunya, West Nile virus in addition to other diseases (Lounibos, O'Meara, & Nishimura, 2003; Santos, Leite, & Falqueto, 2011). However, only 7 of 122 mosquito species reported from bromeliad plants (5.7%) are such disease vectors (Harbach, 2017). In *Guzmania* spp. bromeliads, populations of *Anopheles* spp. and *Culex* spp. can be reduced by consumptive and nonconsumptive effects of damselfly predators (Hammill, Atwood, & Srivastava, 2015). However, these mosquito species can impose strong negative impacts on human populations. Bromeliads can influence diseases that threaten amphibians, such as the fungus *Batrachochytrium dendrobatidis*, which infects and reduces anuran populations throughout the Neotropics. Bromeliads can act as environmental refugia in which the fungus *B. dendrobatidis* has lower prevalence than other ecosystems (Burrowes, Martes, Torres‐Ríos, & Longo, 2017). The high fluctuation in temperature, and other physical and chemical characteristics of bromeliad water, renders this habitat less suitable for the fungus development, which reduces the rates of infection (Blooi et al., 2017; Burrowes et al., 2017). Therefore, the probability of *B. dendrobatidis* infection of frogs in the soil habitats is twice as high as in arboreal microhabitats, such as bromeliads (Burrowes et al., 2017).

#### 3.3.2 Water regulation

The tank bromeliads regulate water dynamics in their tank through the storage of water entering the system as rainfall and fog. The amount of water stored in bromeliad tanks varies according to geographical location, local environmental conditions, and bromeliad abundance and traits. The amount of water per hectare held in tank bromeliads has been estimated to be more than 40,000 L in Brazilian Restinga Forests (Cogliatti‐Carvalho, Rocha‐Pessôa, Nunes‐Freitas, & Rocha, 2010) and over 50,000 L in Colombian cloud forest (Fish, 1983). In addition, the amount of water reserved by bromeliad species range from 8.3 ml to 949.23 ml, but this depends on the bromeliad species and ecosystem type (Appendix S4B). High densities of tank bromeliads may increase water storage, reduce water loss, or affect the water cycle via temporal and spatial redistribution.

Tank bromeliads have a higher water storage capacity than other epiphytes. For that reason, tank bromeliads reduce stemflow and throughflow and then increase water storage inside forests (Van Stan & Pypker, 2015). Moreover, fog interception by bromeliad leaves could increase the total water storage capacity of bromeliads and offset evaporation losses (Guevara‐Escobar et al., 2011; Martorell & Ezcurra, 2007). Plant morphology, including elongated hair‐like structures and rounded formations, enhances bromeliad capacity to retain water (Guevara‐Escobar et al., 2011; Martin & Schmitt, 1989). The number of narrow leaves and the bromeliad size is strongly related to the capacity for water interception (Martorell & Ezcurra, 2007; Zotz & Vera, 1999).

#### 3.3.3 Carbon dioxide (CO_2_) and methane (CH_4_) capture

Bromeliad plants can contribute to climate regulation through the capture and storage of carbon. The absorption of the greenhouse gas CO_2_ through CAM metabolism has been studied in bromeliads, showing that CAM bromeliads are more efficient in carbon uptake than C3 bromeliads (Pierce, Winter, & Griffiths, 2002). Bromeliads contributed 12.8% of the primary net forest productivity of humid forest in Puerto Rico (Richardson et al., 2000). The production of organic matter of bromeliads was 327.8 kg/ha, representing 3.1% of the total organic matter produced in a primary Atlantic Forest of Brazil (Oliveira, 2004) and 910.6 kg/ha in a montane humid forest of Colombia (Isaza, Betancur, & Estévez‐Varón, 2004).

Archaea, methanotrophic bacteria, and invertebrate consumers inhabiting bromeliads also play an important role in the carbon cycle (Atwood et al., 2013; Brandt, Martinson, & Conrad, 2017; Goffredi, Jang, Woodside, & Ussler, 2011). Archaea communities in bromeliad species *Aechmea mariae‐reginae*, *Aechmea nudicaulis*, *Werauhia gladioliflora*, *Werauhia kupperiana*, *Androlepis skinneri*, and *Guzmania lingulata *have been shown to induce methane rates between 12 and 72 nmol CH_4_ ml^−1^ day^−1^ in Costa Rica (Goffredi et al., 2011). In Ecuador, the three functional types of bromeliad ephemeral tank, absorbing trichome tank, and intermediate atmospheric tank bromeliads produce 2.9–37.3 μg CH_4_ L^−1^ (Martinson et al., 2010). Methanotrophic bacteria use methane as a source of energy and reduce methane emissions from bromeliads (Brandt et al., 2017). Cascading impacts of apex predators on bromeliad food webs have been shown to reduce carbon dioxide emissions into the atmosphere. This effect was caused by damselfly predators reducing the biomass of detritivores, which consequently reduce the loss of detritus and release CO_2_ into the atmosphere (Atwood et al., 2013).

### 3.4 Cultural services

The studies that investigated the cultural services provided by bromeliad plants can be further categorized as follows: research about traditional knowledge (4.2%), aesthetic appreciation, (0.97%), and cultural heritage (0.64%) (Figure 2 and Appendix S5).

#### 3.4.1 Traditional knowledge

Traditional knowledge is a source of information about medicinal and food properties of bromeliads and, thus, is closely related to the provisioning services. Ethnobotanical and ethnopharmacological studies reported that at least one bromeliad species is commonly used by several communities and ethnic groups to treat diseases (Agra, Baracho, Nurit, Basílio, & Coelho, 2007; Albertasse, Thomaz, & Andrade, 2010; De Almeida, Rangel, Ramos, & Silva, 2011; Bieski et al., 2012; de Feo & Soria, 2012; Juárez‐Vázquez et al., 2013; Kujawska, Zamudio, & Hilgert, 2012; Nunes et al., 2015; Sreekeesoon & Mahomoodally, 2014). These communities include the Izoceño‐Guaraní community in Bolivia (Bourdy, Michel, & Roca‐Coulthard, 2004), the Amazon coastal community of Marudá in Brazil (Coelho‐Ferreira, 2009), and Barra do Jucu in Brazil (Albertasse et al., 2010) among others. Some other bromeliad species that are important in traditional knowledge include *Bromelia serra* (Bourdy et al., 2004), *Ananas ananassoides* (Coelho‐Ferreira, 2009), *Encholirium spectabile* (Oliveira, Barros, & Moita Neto, 2010), *Ananas comosus* (Bieski et al., 2015; Komlaga et al., 2015), and *Ananas bracteatus* (Samoisy & Mahomoodally, 2016).

#### 3.4.2 Aesthetic appreciation

Bromeliads have great ornamental potential (Acebey et al., 2010; Mielke, Ribeiro do Valle, Poliquesi, & Cuquel, 2009; Vanhoutte, Ceusters, & Proft, 2016). Twelve bromeliad species have been used as ornamental plants in five Latin American countries (Hornung‐Leoni, 2011). It has also been suggested that bromeliads reduce the temperature in the building interiors. Bromeliads planted on the roofs of buildings absorb some solar radiation, use it for photosynthesis, and reflect it back into the atmosphere (Irsyad, Pasek, & Indartono, 2016).

#### 3.4.3 Cultural heritage

Bromeliads, particularly *Ananas comosus, Puya raimondii,* and the genus *Tillandsia*, have been widely used in ceremonial events. In Peru, *Puya raimondii* is used during the celebration of “Fiesta de las Cruces” (Hornung‐Leoni, 2011). Tillandsia species are used for decorating religious celebrations in Mexico; *T. sphaerocephala* are being used for decorating funerals and weddings in Peru; *Aechmea bracteata* are being used in Mexican rituals “Baño de los 7 Días,” in which a mother and her newborn baby take showers in bromeliad water (Echeverri & Román‐Jitdutjaaño, 2011; Hornung‐Leoni, 2011). Ecotourism with the search for bromeliads has been practiced in Veracruz (Mexico) in order to promote education and economic development of local communities (Baltazar Bernal, Zavala Ruiz, Solís Zanotelli, Pérez Sato, & Sánchez Eugenio, 2014).

## 4 DISCUSSION

The Bromeliaceae family provides a diverse array of ecosystem services. The most important services include the maintenance of taxonomic and genetic diversity, provisioning of chemical and pharmaceutical products, food and fiber, traditional knowledge, aesthetic appreciation, cultural heritage, climate control, disease control, and water storage. Bromeliads support high biodiversity by providing resources and serving as microhabitats for other species. Birds and mammals feed on bromeliads or consume the water that they retain (Ferrari & Hilário, 2011; Hayes et al., 2009; Souza, Uetanabaro, Landgref Filho, & Faggioni, 2009). Amphibians, reptiles, odonates, ants, spiders, and other taxa feed on immature life stages of invertebrates associated with bromeliad plants (Appendix S2A).

Most of the recent research works focused on the role of bromeliads in the diversity maintenance of aquatic and terrestrial taxa. Twenty‐five papers reported new species of cyanobacteria, mites, chironomids, protozoa, yeasts, crustaceans, syrphids, psychodids, and salamanders associated with bromeliads. *Bromeliothrix metopoides* (Colpodidae), a ciliate restricted to bromeliads (Foissner, 2010; Weisse, Scheffel, Stadler, & Foissner, 2013), was together with a list of yeast and protist species exclusively found in bromeliads. A cyanobacterium *Brasilonema bromeliae* (Sant'Anna et al., 2011), a smut fungus *Pattersoniomyces tillandsiae* (Piątek et al., 2017), and more than 26 yeast species such as *Kazachstania bromeliacearum*, *K. rupícola*, *Occultifur brasiliensis* (Cystobasidiaceae), *Kockovaella libkindii* (Cuniculitremaceae), *Candida bromeliacearum*, *C. ubatubensis*, *C. intermedia*, *Hagleromyces aurorensis*, *Papiliotrema leoncinii*, *P. miconiae,* and *Cryptococcus albidus* (Tremellaceae) directly depend on bromeliad habitats (Araújo, Medeiros, Mendonça‐Hagler, & Hagler, 1998; Araújo et al., 2012; Gomes et al., 2015; Gomes, Safar, Santos, Lachance, & Rosa, 2016; Hagler et al., 1993; Pagani et al., 2016; Ruivo, Lachance, Rosa, Bacci, & Pagnocca, 2005; Safar, Gomes, Marques, Lachance, & Rosa, 2013; Sousa, Morais, Lachance, & Rosa, 2014). Other taxa that are obligatory inhabitants of bromeliads include chironomids *Stenochironomus atlanticus* (De Pinho, Mendes, & Marcondes, 2005), ostracods of the genus *Elpidium* (Danielopol, Pinto, Gross, Pereira, & Riedl, 2014), arboreal frogs *Phytotriades auratus* (Torresdal, Farrell, & Goldberg, 2017), and spiders of the genus *Cupiennius* (Barth, Seyfarth, Bleckmann, & Schüch, 1988).

The biotic interactions among species govern the structure, function, and services of bromeliad microecosystems. For example, the crab *Armases angustipes* consumes the flowers of bromeliad *Aechmea pectinata*, thereby reducing the frequency of visits by hummingbirds and thus interfering with the pollination of this bromeliad species (Canela & Sazima, 2003). Feces of frogs *Scinax hayii* increases nitrogen concentrations in bromeliads, which enhances photosynthesis of the plant (Romero et al., 2010). Through the maintenance of diverse aquatic food webs, bromeliads can establish easily in nutrient‐poor habitats (Leroy, Carrias, Céréghino, & Corbara, 2015). This is advantageous for the cultivation of bromeliads for food, fiber, chemical, and pharmaceutical products, together with their contribution to cultural services, and also highlights the role of these plants for human society.

Habitat loss, climate change, and invasive insects have caused the loss of bromeliad species in the Neotropics (Cooper et al., 2014; Siqueira Filho & Tabarelli, 2006; Wagner & Zotz, 2018; Zotz et al., 2010). An integral valuation of ecosystem services provided by bromeliads could generate new scientific evidence for decision‐makers in regard to the conservation of tank bromeliads. A special emphasis should be placed on bromeliad species that are already threatened (Appendix S1) or those that contribute to the maintenance of endangered species such as the spectacled bear (*Tremartos ornatus*), the birds *Pipile pipile* and *Crax globulosa,* and the frog *Phytotriades auratus*. The ongoing loss of the bromeliad diversity may compromise ecosystems services directly through the loss of a species or indirectly through the loss of microecosystems that disappear together with the associated organisms. It is critical to recognize that the decline in bromeliad abundance and diversity reaches beyond the effect of removing a single species, as they act as habitats for the entire ecological communities. In fact, bromeliad loss could be considered on par with habitat destruction in their effect on the broader ecosystem structure, function, and services.

This review combines the information about the relative importance of the individual ecosystem services with the information about the research efforts across different Neotropical countries. As individual studies have often focused on one or a narrow set of ecosystem services in a single country, we cannot fully separate the importance from research effort. Nevertheless, this synthesis provides a first comprehensive assessment of the role of the Bromeliaceae family, which has often been used by community ecologists as a model ecosystem but has been rarely evaluated in its own merit. Moreover, this synthesis provides an ecological and sociocultural valuation of the Bromeliaceae family, which together with further quantitative and economic valuation can be an important starting point of an integral evaluation of the role these important plants played in providing goods and benefits for human well‐being (TEEB, 2010).

Understanding the role of bromeliads in the maintenance of biodiversity is essential to improve the comprehensive assessment of ecosystem services and to include often overlooked components of tropical ecosystems in public decision‐making processes. However, the contributions of bromeliads to other ecosystem services, apart from their role as habitats, have been largely understudied in the past. While the number of papers about bromeliads providing supporting services has greatly increased over the last two decades, there has been little research on other services and the potential of bromeliads to the provision of pharmaceutical products and nutritional resources, or to regulate climate through water storage and carbon cycling. These services are of critical importance and remain promising venues for future research. Much of the research on ecosystem services has been performed in Brazil, the country with the highest diversity of bromeliads (Versieux & Wendt, 2007). Research efforts in other Neotropical countries that also have a high bromeliads diversity, such as Colombia, Ecuador, Bolivia, Peru, and Venezuela, are required to overcome currently large knowledge gaps about how this diverse and threatened family of plants, directly and indirectly, benefits human societies.

## CONFLICT OF INTEREST

None declared.

## AUTHORS' CONTRIBUTION

FOB and JVE conceived the ideas; GL compiled the data; GL, FOB, and JVE wrote the first draft of the manuscript; LJ and PK provided comments and important intellectual contribution to the manuscript. All authors gave final approval for publication.

## Data Availability

Data will be archived in the public archive Dryad (http://datadryad.org) https://doi.org/10.5061/dryad.nt288h1.
