# Supplementary material for: Ecosystem services provided by bromeliad plants: A systematic review
Source: Ecol Evol. 2019 May 29;9(12):7360–72. doi: 10.1002/ece3.5296 (PMC6662323; doi:10.1002/ece3.5296)
Supplement: Supplementary file 1 [file ECE3-9-7360-s001.docx]

**APPENDIX 1.** Bromeliads under the threatened species category as critically endangered (CR), Endangered (EN) or Vulnerable (VU). The information was obtained from the IUCN Red List of Threatened Species, version 2018-2. Downloaded on 16 December 2018.

| **Bromeliad species** | **Threat category** | **Native** |
| --- | --- | --- |
| *Aechmea aculeatosepala* | VU | Ecuador |
| *Aechmea biflora* | VU | Ecuador |
| *Aechmea cymosopaniculata* | CR | Venezuela |
| *Aechmea downsiana* | CR | Trinidad and Tobago |
| *Aechmea kentii* | EN | Ecuador |
| *Aechmea kleinii* | EN | Brazil |
| *Aechmea lugoi* | VU | Ecuador |
| *Aechmea manzanaresiana* | EN | Ecuador |
| *Aechmea patriciae* | VU | Ecuador |
| *Aechmea roeseliae* | VU | Ecuador |
| *Aechmea tayoensis* | EN | Ecuador |
| *Aechmea wuelfinghoffii* | VU | Ecuador |
| *Bromelia nidus-puellae* | EN | Colombia |
| *Cryptanthus diamantinensis* | CR | Brazil |
| *Cryptanthus pseudoscaposus* | EN | Brazil |
| *Deuterocohnia chrysantha* | VU | Chile |
| *Dyckia reitzii* | EN | Brazil |
| *Encholirium luxor* | EN | Brazil |
| *Greigia atrobrunnea* | VU | Ecuador |
| *Guzmania aequatorialis* | VU | Ecuador |
| *Guzmania albescens* | EN | Ecuador |
| *Guzmania alborosea* | VU | Ecuador |
| *Guzmania alcantareoides* | EN | Ecuador |
| *Guzmania andreettae* | VU | Ecuador |
| *Guzmania atrocastanea* | VU | Ecuador |
| *Guzmania bergii* | VU | Ecuador |
| *Guzmania bismarckii* | CR | Peru |
| *Guzmania condorensis* | EN | Ecuador |
| *Guzmania corniculata* | VU | Ecuador |
| *Guzmania dalstroemii* | VU | Ecuador |
| *Guzmania ecuatorensis* | EN | Ecuador |
| *Guzmania fuerstenbergiana* | EN | Ecuador |
| *Guzmania fuquae* | EN | Ecuador |
| *Guzmania fusispica* | VU | Ecuador |
| *Guzmania harlingii* | VU | Ecuador |
| *Guzmania henniae* | EN | Ecuador |
| *Guzmania hirtzii* | VU | Ecuador |
| *Guzmania hollinensis* | VU | Ecuador |
| *Guzmania inexpectata* | VU | Ecuador |
| *Guzmania izkoi* | VU | Ecuador |
| *Guzmania kentii* | VU | Ecuador |
| *Guzmania lepidota* | CR | Ecuador |
| *Guzmania madisonii* | VU | Ecuador |
| *Guzmania osyana* | EN | Ecuador |
| *Guzmania poortmanii* | CR | Ecuador |
| *Guzmania pseudospectabilis* | VU | Ecuador |
| *Guzmania puyoensis* | VU | Ecuador |
| *Guzmania roseiflora* | EN | Ecuador |
| *Guzmania rubrolutea* | EN | Ecuador |
| *Guzmania sieffiana* | VU | Ecuador |
| *Guzmania striata* | CR | Ecuador |
| *Guzmania zakii* | VU | Ecuador |
| *Hechtia melanocarpa* | VU | Mexico |
| *Hohenbergiopsis guatemalensis* | EN | Guatemala, Mexico |
| *Mezobromelia brownii* | VU | Ecuador |
| *Mezobromelia fulgens* | EN | Ecuador |
| *Navia sandwithii* | VU | Guyana |
| *Neoregelia azevedoi* | EN | Brazil |
| *Pepinia alexanderi* | EN | Ecuador |
| *Pepinia carnososepala* | VU | Ecuador |
| *Pepinia fulgens* | VU | Ecuador |
| *Pepinia harlingii* | VU | Ecuador |
| *Pepinia hooveri* | EN | Ecuador |
| *Pepinia verrucosa* | VU | Ecuador |
| *Pitcairnia aequatorialis* | EN | Ecuador |
| *Pitcairnia alata* | EN | Ecuador |
| *Pitcairnia andreetae* | VU | Ecuador |
| *Pitcairnia bergii* | EN | Ecuador |
| *Pitcairnia caduciflora* | EN | Ecuador |
| *Pitcairnia clarkii* | EN | Ecuador |
| *Pitcairnia devansayana* | VU | Ecuador |
| *Pitcairnia elliptica* | CR | Ecuador |
| *Pitcairnia feliciana* | EN | Guinea |
| *Pitcairnia ferrell-ingramiae* | VU | Ecuador |
| *Pitcairnia hirtzii* | VU | Ecuador |
| *Pitcairnia jareckii* | EN | Puerto Rico |
| *Pitcairnia lutescens* | EN | Ecuador |
| *Pitcairnia oblongifolia* | EN | Ecuador |
| *Pitcairnia oranensis* | CR | Argentina |
| *Pitcairnia poeppigiana* | EN | Peru |
| *Pitcairnia prolifera* | VU | Ecuador |
| *Pitcairnia reflexiflora* | EN | Ecuador |
| *Pitcairnia stevensonii* | VU | Ecuador |
| *Pitcairnia unilateralis* | VU | Ecuador |
| *Pitcairnia violascens* | VU | Ecuador |
| *Puya alata* | VU | Bolivia |
| *Puya angelensis* | EN | Ecuador |
| *Puya castellanosii* | EN | Argentina |
| *Puya compacta* | CR | Ecuador |
| *Puya exigua* | CR | Ecuador |
| *Puya herrerae* | VU | Bolivia, Peru |
| *Puya obconica* | VU | Ecuador |
| *Puya parviflora* | EN | Ecuador |
| *Puya pichinchae* | VU | Ecuador |
| *Puya pygmaea* | VU | Ecuador |
| *Puya raimondii* | EN | Bolivia, Peru |
| *Puya reflexiflora* | VU | Peru |
| *Puya roseana* | EN | Ecuador |
| *Puya sodiroana* | VU | Ecuador |
| *Racinaea blassii* | VU | Ecuador |
| *Racinaea euryelytra* | VU | Ecuador |
| *Racinaea hauggiae* | VU | Ecuador |
| *Racinaea inconspicua* | EN | Ecuador |
| *Racinaea tandapiana* | VU | Ecuador |
| *Racinaea tripinnata* | EN | Ecuador |
| *Ronnbergia campanulata* | EN | Ecuador |
| *Tillandsia acosta-solissi* | EN | Ecuador |
| *Tillandsia aequatorialis* | VU | Ecuador |
| *Tillandsia brenneri* | VU | Ecuador |
| *Tillandsia candida* | VU | Brazil |
| *Tillandsia cernua* | EN | Ecuador |
| *Tillandsia cucullata* | VU | Ecuador |
| *Tillandsia demissa* | EN | Ecuador |
| *Tillandsia dyeriana* | CR | Ecuador |
| *Tillandsia emergens* | VU | Ecuador |
| *Tillandsia hemkeri* | VU | Ecuador |
| *Tillandsia hirtzii* | VU | Ecuador |
| *Tillandsia homostachya* | EN | Ecuator |
| *Tillandsia indigofera* | EN | Ecuador |
| *Tillandsia marnieri-lapostollei* | VU | Ecuador |
| *Tillandsia nervisepala* | EN | Ecuador |
| *Tillandsia ortgiesiana* | VU | Mexico |
| *Tillandsia pachyaxon* | EN | Ecuador |
| *Tillandsia polyantha* | EN | Ecuador |
| *Tillandsia portillae* | EN | Ecuador |
| *Tillandsia pretiosa* | VU | Ecuador |
| *Tillandsia pseudomontana* | EN | Brazil |
| *Tillandsia raackii* | VU | Ecuador |
| *Tillandsia rhodosticta* | VU | Ecuador |
| *Tillandsia rubroviolacea* | EN | Ecuador |
| *Tillandsia sodiroi* | VU | Ecuador |
| *Tillandsia umbellata* | EN | Ecuador |
| *Tillandsia yerbasantae* | CR | Mexico |
| *Tillandsia zarumensis* | EN | Ecuador |
| *Vriesea andreettae* | VU | Ecuador |
| *Vriesea appendiculata* | EN | Ecuador |
| *Vriesea camptoclada* | VU | Costa Rica |
| *Vriesea drewii* | EN | Ecuador |
| *Vriesea limonensis* | VU | Ecuador |
| *Vriesea lutheri* | VU | Ecuador |
| *Vriesea penduliscapa* | VU | Ecuador |
| *Vriesea petraea* | EN | Ecuador |
| *Vriesea strobeliae* | EN | Ecuador |
| *Vriesea tillii* | VU | Ecuador |
| *Vriesea wuelfinghoffii* | VU | Ecuador |
| *Werauhia diantha* | EN | Ecuador |
| *Werauhia haltonii* | VU | Ecuador |
| *Werauhia paupera* | VU | Ecuador |
| *Wittmackia caymanensis* | CR | Cayman Islands |
