# Supplementary material for: Ecosystem services provided by bromeliad plants: A systematic review
Source: Ecol Evol. 2019 May 29;9(12):7360–72. doi: 10.1002/ece3.5296 (PMC6662323; doi:10.1002/ece3.5296)
Supplement: Supplementary file 2 [file ECE3-9-7360-s002.docx]

**APPENDIX 2. Supporting services provided by bromeliads.**

**Appendix 2A.** Fauna inhabiting tank bromeliads and how they use these plants.

| **Taxonomic Group** | **Use** | **References** |
| --- | --- | --- |
| Annelids:  Oligochaete | Habitat | Fragoso & Rojas-Fernandez, 1996; Schmelz *et al*., 2015 |
| Insects: Diptera, Coleoptera, Lepidoptera, Hemiptera, Hymenoptera | Habitat and food (Nectar, pollen and leaves in larval stages) | Aguilar-Rodríguez *et al*., 2016; Balke *et al*., 2008; Bernardello *et al*., 1991; Burmeister, 1985; Cardoso *et al*., 2015; Céréghino *et al*., 2010; Céréghino *et al*., 2011; Ceretti-Junior *et al*., 2016; Christianini *et al*., 2013; Clarkson *et al*., 2014; Cranston, 2007; Epler & Janetzky, 1998; Frank & Lounibos, 2009; Fumero-Cabán & Meléndez-Ackerman, 2007; Greeney, 2001; Greeney, 2014; Grishin & Durden, 2012; Jabiol *et al*., 2009; Jocque & Field, 2014; Judd, 1998; Leroy *et al*., 2009a; Leroy *et al*., 2011; Leroy *et al*., 2013; Liria, 2007; Marques & Forattini, 2009; Marques *et al*., 2012; Marteis *et al*., 2017; Mestre *et al*., 2001; Montero *et al*. 2010; Morales *et al*., 2017; Moura *et al*., 2006; Ngai & Srivastava, 2006; Ospina-Bautista *et al*., 2008; Pereira *et al*., 2007; Robbins & Nicolay, 2001; Robbins, 2010; Rocca & Sazima, 2013; Rotheray *et al*., 2007; Santana & Machado, 2010; Schmidt & Zotz, 2000; Schmid *et al*., 2011a; Schmid *et al*., 2011b; Sodré *et al*., 2010; Starzomski *et al*., 2010; Srivastava *et al*., 2005; Torreias *et al*., 2008; Torreias *et al*., 2010; Torreias & Ferreira-Keppler, 2011; Wagner *et al*., 2010; Wendt *et al*., 2001; Wittman, 2000; Zillikens *et al*., 2001 |
| Arachnids: Spiders, opiliones, mites | Habitat | Barth *et al*., 1988; De Omena & Romero, 2008; Gonçalves-Souza *et al*., 2010; Gonçalves *et al*., 2011; Nesbitt, 1985; Osses *et al*., 2008; Pešić *et al*., 2015; Pešić *et al*., 2016; Romero *et al*., 2006; Romero *et al*., 2008. |
| Crustaceans: Crabs, ostracods | Habitat and food (Inflorescences) | Canela & Sazima, 2003; Danielopol *et al*., 2014; Diesel, 1989; Diesel, 1992; Diesel & Schuh, 1993; Diesel, 1997; Ospina-Bautista *et al*., 2016; Smirvov, 1988; Wehrtmann *et al*., 2016. |
| Amphibians: Anurans, salamanders | Habitat | Bourne *et al*., 2001; Brito *et al*., 2017; Catenazzi & Lehr, 2009; Cunha & Napoli, 2016; Dabés *et al*., 2012; Domingos *et al*., 2015; Feder, 1982; Ferreira *et al*., 2012; Ferreira *et al*., 2015; Haddad & Pombal, 1998; Jungfer, 1996; Jungfer & Weygoldt, 1999; Mageski *et al*., 2016; Moura *et al*., 2011; Pederassi *et al*., 2012; Poelman & Dicke, 2008; Poelman *et al*., 2013; Pontes *et al*., 2013; Romero *et al*., 2010; Ruano-Fajardo *et al*., 2014; Sabagh *et al*., 2012; Silva *et al*., 2011; Teixeira *et al*., 1997; Teixeira *et al*., 2002; Torresdal *et al*., 2017; Vásquez-Almazán *et al*., 2009; Weygoldt, 1989. |
| Reptiles: Lizards | Habitat | Henle & Knogge, 2009; Vrcibradic & Rocha, 1996. |
| Birds | Habitat, source of drinking water and food (Nectar and fruits) | Alves *et al*., 2002; Bernardello *et al*., 1991; Buzato *et al*., 2001; Canela & Sazima, 2003; Canela & Sazima, 2005; Cestari & Pizo, 2008; De Queiroz Piacentini & Varassin, 2007; Dziedzioch *et al*., 2003; Fumero-Cabán & Meléndez-Ackerman, 2007; González-Gómez & Valdivia, 2005; Hayes *et al*., 2009; Hornung-Leoni *et al*., 2013; Leal *et al*., 2006; Leite *et al*., 2017; Pansarin & Pedro, 2016;Ríos & Cascante-Marín, 2017; Rocca & Sazima, 2013; Santana & Machado, 2010; Sazima *et al*., 1995; Sazima *et al*., 1996; Sazima & Sazima, 1999; Schmid *et al*., 2011a; Siquiera Filho & Machado, 2001; Souza *et al*., 2009. |
| Mammals:  Rodents, bats, coati, peccaries, primates, spectacled bear | Food (Leaves, inflorescences, nectar, pollen, stems, succulent heart), Source of drinking water | Aguilar-Rodríguez *et al*., 2016; Amora *et al*., 2013; Beisiegel, 2001; Brown & Zunino, 1990; Catenacci, 2016; Demay *et al*., 2014; Ferrari & Hilário, 2011; Gonçalves-Oliveira *et al*., 2017;  Hmeljevski *et al*., 2017; Sazima *et al*., 1989; Sazima *et al*., 1999; Suárez, 1988; Rosati & Bucher, 1992; Ticktin, 2003; Tschapka & Von Helversen, 2007; Wendt *et al*., 2001. |

**Appendix 2B.** Contribution of bromeliads for Nutrient cycling. (I) as a microecosystem that forms a habitat for microorganisms, aquatic invertebrates, and some vertebrate species or (II) as organism in themselves.

| **Contribution as** | **References** |
| --- | --- |
| I | Atwood *et al*., 2013; Atwood *et al*., 2014; Brandt *et al*., 2015; Brandt *et al*., 2017; Breviglieri *et al*., 2017; Breviglieri & Romero, 2017; Brouard *et al*., 2012; Carrias *et al*., 2001; Castaño-Meneses *et al*., 2014; Farjalla *et al*., 2016; Gonçalves *et al*., 2014; González *et al*., 2011; Haubrich *et al*., 2009; Inselsbacher *et al*., 2007; Klann *et al*., 2016; Kotowska & Werner, 2013; Leroy *et al*., 2009 a,b; Leroy *et al*., 2011; Leroy *et al*., 2013; Leroy *et al*., 2015; Leroy *et al*., 2017; Marino *et al*., 2011; Marino *et al*., 2016; Monteiro & Macedo, 2014; Pett-Ridge & Silver, 2002; Płachno *et al*., 2017; Richardson *et al*., 2000 a,b; Romero *et al*., 2008; Romero *et al*., 2010; Souza *et al*., 2016; Suleiman *et al*., 2017; Talaga *et al*., 2015. |
| II | De Oliveira, 2004; De Omena *et al*., 2017; Gonçalves *et al*., 2011; Looby *et al*., 2012; Ngai & Srivastava, 2006. |

**REFERENCES**

Aguilar-Rodríguez, P. A., Krömer, T., García‐Franco, J. G., & MacSwiney, G. (2016). From dusk till dawn: nocturnal and diurnal pollination in the epiphyte *Tillandsia heterophylla* (Bromeliaceae). *Plant Biology*, 18 (1), 37-45.

Alves, M. A., Rocha, C. F. D., Van Sluys, M., & Vecchi, M. (2002). Nest, eggs and effort partitioning in incubation and rearing by a pair of the Black-cheecked gnateater, *Conopophaga melanops* (Passeriformes, Conopophagidae), in an Atlantic Rainforest area of Rio de Janeiro, Brazil. *Revista Brasileira de Ornitologia-Brazilian*, 10 (1), 67-71.

Amora, T. D., Beltrão‐Mendes, R. A. O. N. E., & Ferrari, S. F. (2013). Use of Alternative Plant Resources by Common Marmosets (*Callithrix jacchus*) in the Semi‐Arid Caatinga Scrub Forests of Northeastern Brazil. *American Journal of Primatology*, 75 (4), 333-341.

Atwood, T. B., Hammill, E., Greig, H. S., Kratina, P., Shurin, J. B., Srivastava, D. S., & Richardson, J. S. (2013). Predator-induced reduction of freshwater carbon dioxide emissions. *Nature Geoscience*, 6 (3), 191-194.

Atwood, T. B., Hammill, E., Srivastava, D. S., & Richardson, J. S. (2014). Competitive displacement alters top-down effects on carbon dioxide concentrations in a freshwater ecosystem. *Oecologia*, 175 (1), 353-361.

Balke, M., Gómez-Zurita, J., Ribera, I., Viloria, A., Zillikens, A., Steiner, J., Garcia, M., Hendrich, L., & Vogler, A. (2008). Ancient associations of aquatic beetles and tank bromeliads in the Neotropical forest canopy. *Proceedings of the National Academy of Sciences,* 105 (17), 6356-6361.

Barth, F. G., Seyfarth, E. A., Bleckmann, H., & Schüch, W. (1988). Spiders of the genus *Cupiennius* Simon 1891 (Araneae, Ctenidae). I. Range distribution, dwelling plants, and climatic characteristics of the habitats. *Oecologia*, 77(2), 187-193.

Beisiegel, B. M. (2001). Notes on the coati, *Nasua nasua* (Carnivora: Procyonidae) in an Atlantic forest area. *Brazilian Journal of Biology*, 61 (4), 689-692.

Bernardello, M., Galetto, L., & Juliani, H. R. (1991). Floral nectar, nectary structure and pollinators in some Argentinean Bromeliaceae. *Annals of Botany*, 67 (5), 401-411.

Bourne, G. R., Collins, A. C., Holder, A. M., & McCarthy, C. L. (2001). Vocal communication and reproductive behavior of the frog *Colostethus beebei* in Guyana. *Journal of Herpetology*, 35 (2), 272-281.

Brandt, F. B., Martinson, G. O., & Conrad, R. (2017). Bromeliad tanks are unique habitats for microbial communities involved in methane turnover. *Plant and Soil*, 410 (1-2), 167-179.

Brandt, F. B., Martinson, G. O., Pommerenke, B., Pump, J., & Conrad, R. (2015). Drying effects on archaeal community composition and methanogenesis in bromeliad tanks. *FEMS Microbiology Ecology*, 91, 1-10.

Breviglieri, C. P. B., & Romero, G. Q. (2017). Terrestrial vertebrate predators drive the structure and functioning of aquatic food webs. *Ecology*, 98 (8), 2069-2080.

Breviglieri, C. P. B., Oliveira, P. S., & Romero, G. Q. (2017). Fear mediates trophic cascades: nonconsumptive effects of predators drive aquatic ecosystem function. *The American Naturalist,* 189 (5), 490-500.

Brito, J., Almendáriz, A., Batallas, D., & Ron, S. R. (2017). Nueva especie de rana bromelícola del género Pristimantis (Amphibia: Craugastoridae), meseta de la Cordillera del Cóndor, Ecuador. *Papéis Avulsos de Zoologia*, 57(15), 177-195.

Brouard, O., Céréghino, R., Corbara, B., Leroy, C., Pelozuelo, L., Dejean, A., & Carrias, J. F. (2012). Understorey environments influence functional diversity in tank‐bromeliad ecosystems. *Freshwater Biology*, 57 (4), 815-823.

Brown, A. D., & Zunino, G. E. (1990). Dietary variability in *Cebus apella* in extreme habitats: evidence for adaptability. *Folia primatologica*, 54 (3-4), 187-195.

Burmeister, EG. (1985). Bromeliáceas como espacio vital para larvas de Conocephalidae y Copiphoridae (Insecta, Saltatoria, Ensifera, Tettigonioidea). *Studies on Neotropical Fauna and Environment*, 20, 107– 111.

Buzato, S., Sazima, M., & Sazima, I. (2001). Hummingbird‐Pollinated Floras at Three Atlantic Forest Sites. *Biotropica*, 32(4b), 824-841.

Canela, M. B. F., & Sazima, M. (2003). *Aechmea pectinata*: a Hummingbird‐dependent Bromeliad with Inconspicuous Flowers from the Rainforest in South‐eastern Brazil. *Annals of Botany*, 92(5), 731-737.

Canela, M. B. F., & Sazima, M. (2005). The pollination of *Bromelia antiacantha* (Bromeliaceae) in Southeastern Brazil: ornithophilous versus melittophilous features. *Plant Biology*, 7(4), 411-416.

Cardoso, C. A. A., Lourenço-de-Oliveira, R., Codeço, C. T., & Motta, M. A. (2015). Mosquitoes in bromeliads at ground level of the Brazilian Atlantic Forest: the relationship between mosquito fauna, water volume, and plant type. *Annals of the Entomological Society of America*, 108(4), 449-458.

Carrias, J. F., Cussac, M. E., & Corbara, B. (2001). A preliminary study of freshwater protozoa in tank bromeliads. *Journal of Tropical Ecology*, 17(4), 611-617.

Castaño-Meneses, G., Mercado, I., García-Calderón, N., & Palacios-Vargas, J.G. (2014). Correlation between arthropods and physical and chemical characteristics of water and soil retained in *Tillandsia violacea* (bromeliaceae) in an Abies-Quercus forest in central Mexico. *Applied Ecology and Environmental Research*, 12(1), 179-192.

Catenacci, L. S., Pessoa, M. S., Nogueira-Filho, S. L., & De Vleeschouwer, K. M. (2016). Diet and feeding behavior of *Leontopithecus chrysomelas* (Callitrichidae) in degraded areas of the Atlantic forest of South-Bahia, Brazil. *International Journal of Primatology*, 37(2), 136-157.

Catenazzi, A., & Lehr, E. (2009). The generic allocation of “Hyla” antoniiochoai De la Riva & Chaparro, 2005 (Anura), with description of its advertisement call and ecology. *Zootaxa*, 2304, 61-68.

Céréghino, R., Leroy, C., Dejean, A., & Corbara, B. (2010). Ants mediate the structure of phytotelm communities in an ant‐garden bromeliad. *Ecology*, 91(5), 1549-1556.

Céréghino, R., Leroy, C., Carrias, J. F., Pelozuelo, L., Segura, C., Bosc, C., Dejean, A., & Corbara, B. (2011). Ant–plant mutualisms promote functional diversity in phytotelm communities. *Functional Ecology*, 25(5), 954-963

Ceretti-Junior, W., de Oliveira Christe, R., Rizzo, M., Strobel, R. C., de Matos Junior, M. O., de Mello, M. H. S. H., M. H., Fernandes, A., Medeiros-Sousa, A. R., deCarvalho, G. C, & Marrelli, M. T. (2016). Species composition and ecological aspects of immature mosquitoes (Diptera: Culicidae) in bromeliads in urban parks in the city of Sao Paulo, Brazil. *Journal of arthropod-borne diseases*, 10(1), 102-112.

Cestari, C., & Pizo, M. A. (2008). Utilization of epiphytes by birds in a Brazilian Atlantic forest. *Ornitologia Neotropical*, 19, 97-107.

Christianini, A. V., Forzza, R. C., & Buzato, S. (2013). Divergence on floral traits and vertebrate pollinators of two endemic Encholirium bromeliads. *Plant Biology*, 15 (2), 360-368.

Clarkson, B., Albertoni, F. F., & Fikáček, M. (2014). Taxonomy and biology of the bromeliad-inhabiting genus Lachnodacnum (Coleoptera: Hydrophilidae: Sphaeridiinae). *Acta Entomologica Musei Nationalis Pragae*, 54 (1), 157-194.

Cranston, P. S. (2007). A new species for a bromeliad phytotelm-dwelling Tanytarsus (Diptera: Chironomidae). *Annals of the Entomological Society of America*, 100 (5),617-622.

Cunha, M. S., & Napoli, M. F. (2016). Calling site selection by the bromeliad-dwelling treefrog *Phyllodytes melanomystax* (Amphibia: Anura: Hylidae) in a coastal sand dune habitat. *Studies on neotropical fauna and environment*, 51 (2), 144-151.

Dabés, L., Bonfim, V. M. G., Napoli, M. F., & Klein, W. (2012). Water balance and spatial distribution of an anuran community from Brazil. *Herpetologica*, 68 (4), 443-455.

Danielopol, D. L., Pinto, R. L., Gross, M., Pereira, J. D. S., & Riedl, N. (2014). On the evolutionary biology of Elpidium ostracods (Limnocytheridae, Timiriaseviinae): A proposal for pluridisciplinary studies. *Geo-eco-marina*, 20, 87-129.

De Oliveira, R.R. (2004). The importance of epiphytic bromeliads on the turnover of nutrients at the Atlantic Rain Forest. *Acta Botanica Brasilica*, 18 (4), 793-799.

De Omena, P. M., & Romero, G. Q. (2008). Fine-scale microhabitat selection in a bromeliad-dwelling jumping spider (Salticidae). *Biological journal of the Linnean Society*, 94 ( 4), 653-662.

De Omena, P. M., Srivastava, D. S., & Romero, G. Q. (2017). Does the strength of cross-ecosystem trophic cascades vary with ecosystem size? A test using a natural microcosm. *Freshwater biology*, 62 (4), 724-736.

De Queiroz Piacentini, V., & Varassin, I. G. (2007). Interaction network and the relationships between bromeliads and hummingbirds in an area of secondary Atlantic rain forest in southern Brazil. *Journal of Tropical Ecology*, 23 (6), 663-671.

Demay, S. M., Roon, D. A., Rachlow, J. L., & Cisneros, R. (2014). Selective foraging on bromeliads by Andean bears in the Ecuadorian páramo. *Ursus*, 25 (2), 139-147.

Diesel, R. (1989). Parental care in an unusual environment: *Metopaulias depressus* (Decapoda: Grapsidae), a crab that lives in epiphytic bromeliads. *Animal Behaviour*, 38 (4), 561-575.

Diesel, R. (1992). Managing the offspring environment: brood care in the bromeliad crab, *Metopaulias depressus*. *Behavioral Ecology and Sociobiology*, 30 (2), 125-134.

Diesel, R. (1997). Maternal control of calcium concentration in the larval nursery of the bromeliad crab, *Metopaulias depressus* (Grapsidae). *Proceedings of the Royal Society of London. Series B: Biological Sciences*, 264 (1387), 1403-1406.

Diesel, R., & Schuh, M. (1993). Maternal care in the bromeliad crab *Metopaulias depressus* (Decapoda): maintaining oxygen, pH and calcium levels optimal for the larvae. Behavioral *Ecology and Sociobiology*, 32 (1), 11-15.

Domingos, F. M. C. B., Arantes, I. C., Cavalcanti, D. R., & Jotta, P. A. C. V. (2015). Shelter from the sand: microhabitat selection by the bromelicolous tree frog *Scinax cuspidatus* (Anura, Hylidae) in a Brazilian restinga. *Northwest Journal of Zoology*, 11, 27-33.

Dziedzioch, C., Stevens, A. D., & Gottsberger, G. (2003). The hummingbird plant community of a tropical montane rain forest in southern Ecuador. *Plant Biology*, 5(3), 331-337.

Epler, J. H., & Janetzky, W. J. (1998). A new species of Monopelopia (Diptera: Chironomidae) from phytotelmata in Jamaica, with preliminary ecological notes. *Journal of the Kansas* *Entomological Society*, 71 (3), 216-225.

Farjalla, V. F., González, A. L., Céréghino, R., Dézerald, O., Marino, N. A., Piccoli, G. C.,Richardson, B.A., Richardson, M.J.,Romero, G.Q., & Srivastava, D. S. (2016). Terrestrial support of aquatic food webs depends on light inputs: a geographically‐replicated test using tank bromeliads. *Ecology*, 97 (8), 2147-2156.

Fragoso, C., & Rojas-Fernandez, P. (1996). Earthworms inhabiting bromeliads in Mexican tropical rainforests: ecological and historical determinants. *Journal of tropical ecology*, 12 (5), 729-734.

Feder, M. E. (1982). Thermal ecology of neotropical lungless salamanders (Amphibia: Plethodontidae): environmental temperatures and behavioral responses. *Ecology*, 63 (6), 1665-1674.

Ferrari, S. F., & Hilário, R. R. (2011). Use of water sources by buffy-headed marmosets (*Callithrix flaviceps*) at two sites in the Brazilian Atlantic Forest. *Primates*, 53 (1),65-70.

Ferreira, R. B., Schineider, J. A., & Teixeira, R. L. (2012). Diet, fecundity, and use of bromeliads by *Phyllodytes luteolus* (Anura: Hylidae) in southeastern Brazil. *Journal of Herpetology*, 46 (1), 19-24.

Ferreira, R. B., Faivovich, J., Beard, K. H., & Pombal Jr, J. P. (2015). The First Bromeligenous Species of Dendropsophus (Anura: Hylidae) from Brazil's Atlantic Forest. *PloS one*, 10 (12), e0142893.

Frank, J. H., & Lounibos, L. P. (2009). Insects and allies associated with bromeliads: a review. *Terrestrial arthropod reviews*, 1 (2), 125-153.

Fumero‐Cabán, J. J., & Meléndez‐Ackerman, E. J. (2007). Relative pollination effectiveness of floral visitors of *Pitcairnia angustifolia* (Bromeliaceae). *American Journal of botany*, 94 (3), 419-424.

Gonçalves-Souza, T., Brescovit, A. D., Rossa-Feres, D. D. C., & Romero, G. Q. (2010). Bromeliads as biodiversity amplifiers and habitat segregation of spider communities in a Neotropical rainforest. *Journal of Arachnology*, 38 (2), 270-279.

Gonçalves, A. Z., Hoffmann, F. L., Mercier, H., Mazzafera, P., & Romero, G. Q. (2014). Phyllosphere bacteria improve animal contribution to plant nutrition. *Biotropica*, 46 (2), 170-174.

Gonçalves, A. Z., Mercier, H., Mazzafera, P., & Romero, G. Q. (2011). Spider-fed bromeliads: seasonal and interspecific variation in plant performance. *Annals of Botany*, 107 (6), 1047-1055.

Gonçalves‐Oliveira, R. C., Wöhrmann, T., Benko‐Iseppon, A. M., Krapp, F., Alves, M., Wanderley, M. D. G. L., & Weising, K. (2017). Population genetic structure of the rock outcrop species *Encholirium spectabile* (Bromeliaceae): The role of pollination vs. seed dispersal and evolutionary implications. *American Journal of Botany*, 104 (6), 868-878.

González‐Gómez, P. L., & Valdivia, C. E. (2005). Direct and Indirect Effects of Nectar Robbing on the Pollinating Behavior of *Patagona gigas* (Trochilidae) 1. *Biotropica:* *The Journal of Biology and Conservation*, 37(4), 693-696.

González, A. L., Fariña, J. M., Pinto, R., Pérez, C., Weathers, K. C., Armesto, J. J., & Marquet, P. A. (2011). Bromeliad growth and stoichiometry: responses to atmospheric nutrient supply in fog-dependent ecosystems of the hyper-arid Atacama Desert, Chile. *Oecologia*, 167(3), 835-845.

Greeney, H. F. (2001). The insects of plant-held waters: a review and bibliography*. Journal of Tropical Ecology,* 17(2), 241-260.

Greeney, H. F. (2014). Breeding biology of the Grey-breasted Flycatcher *Lathrotriccus griseipectus* in south-west Ecuador. *Bulletin of the British Ornithologists’Club*, 134, 14-18.

Grishin, N. V., & Durden, C. J. (2012). New Bromeliad-feeding Strymon species from Big Bend National Park, Texas, USA and its vicinity (Lycaenidae: Theclinae). *The Journal of the Lepidopterists' Society*, 66 (2), 81-110.

Haddad, C. F., & Pombal Jr, J. P. (1998). Redescription of *Physalaemus spiniger* (Anura: Leptodactylidae) and description of two new reproductive modes. *Journal of Herpetology*, 32 (4), 557-565.

Haubrich, C. S., Pires, A. P., Esteves, F. A., & Farjalla, V. F. (2009). Bottom-up regulation of bacterial growth in tropical phytotelm bromeliads. *Hydrobiologia*, 632(1), 347-353.

Hayes, F. E., Shameerudeen, C. L., Sanasie, B., Hayes, B. D., Ramjohn, C. L., & Lucas, F. B. (2009). Ecology and behaviour of the critically endangered Trinidad piping-guan *Aburria pipile*. *Endangered Species Research*, 6 (3), 223-229.

Henle, K., & Knogge, C. (2009). Water-filled bromeliad as roost site of a tropical lizard, *Urostrophus vautieri* (Sauria: Leiosauridae). *Studies on Neotropical Fauna and Environment*, 44 (3), 161-162.

Hmeljevski, K. V., Wolowski, M., Forzza, R. C., & Freitas, L. (2017). High outcrossing rates and short-distance pollination in a species restricted to granitic inselbergs. *Australian journal of botany*, 65 (4), 315-326.

Hornung-Leoni, C. T., González-Gómez, P. L., & Troncoso, A. J. (2013). Morphology, nectar characteristics and avian pollinators in five Andean Puya species (Bromeliaceae). *Acta Oecologica,* 51, 54-61.

Inselsbacher, E., Cambui, C. A., Richter, A., Stange, C. F., Mercier, H., & Wanek, W. (2007). Microbial activities and foliar uptake of nitrogen in the epiphytic bromeliad *Vriesea gigantea*. *New Phytologist*, 175 (2), 311-320.

Jabiol, J., Corbara, B., Dejean, A., & Céréghino, R. (2009). Structure of aquatic insect communities in tank-bromeliads in an East-Amazonian rainforest in French Guiana. Forest *Ecology and Management*, 257 (1), 351-360.

Jocque, M., & Field, R. (2014). Aquatic invertebrate communities in tank bromeliads: how well do classic ecological patterns apply?. *Hydrobiologia*, 730 (1), 153-166.

Judd, D. D. (1998). Review of a bromeliad-ovipositing lineage in Wyeomyia and the resurrection of Hystatomyia (Diptera: Culicidae). *Annals of the Entomological Society of America*, 91 (5), 572-589.

Jungfer, K. H. (1996). Reproduction and parental care of the coronated treefrog, Anotheca spinosa (Steindachner, 1864) (Anura: Hylidae). *Herpetologica*, 44 (1), 55-57.

Jungfer, K. H., & Weygoldt, P. (1999). Biparental care in the tadpole-feeding Amazonian treefrog *Osteocephalus oophagus*. *Amphibia-Reptilia*, 20 (3), 235-249.

Klann, J., McHenry, A., Montelongo, C., & Goffredi, S. K. (2016). Decomposition of plant sourced carbon compounds by heterotrophic betaproteobacteria isolated from a tropical Costa Rican bromeliad. *Microbiology Open*, 5 (3), 479-489.

Kotowska, M. M., & Werner, F. A. (2013). Environmental controls over methane emissions from bromeliad phytotelmata: The role of phosphorus and nitrogen availability, temperature, and water content. *Global Biogeochemical Cycles*, 27 (4), 1186-1193.

Leal, F. C., Lopes, A. V., & Machado, I. C. (2006). Pollination by hummingbirds in a" caatinga" area in Pernambuco State, northeastern Brazil. *Brazilian Journal of Botany*, 29 (3), 379-389.

Leite, G. A., Farias, I. P., Peres, C. A., & Brooks, D. M. (2017). Reproductive biology of the endangered wattled curassow (*Crax globulosa*; Galliformes: Cracidae) in the Juruá River Basin, Western Brazilian Amazonia. *Journal of Natural History*, 51 (11-12), 677-687.

Leroy, C., Corbara, B., Dejean, A., & Céréghino, R. (2009a). Ants mediate foliar structure and nitrogen acquisition in a tank‐bromeliad. *New Phytologist*, 183 (4), 1124-1133.

Leroy, C., Corbara, B., Dejean, A., & Céréghino, R. (2009b). Potential sources of nitrogen in an ant-garden tank-bromeliad. *Plant Signaling & Behavior*, 4 (9), 868-870.

Leroy, C., Corbara, B., Pélozuelo, L., Carrias, J. F., Dejean, A., & Céréghino, R. (2011). Ant species identity mediates reproductive traits and allocation in an ant-garden bromeliad. *Annals of Botany*, 109 (1), 145-152.

Leroy, C., Carrias, J. F., Corbara, B., Pélozuelo, L., Dézerald, O., Brouard, O., Dejean, A., & Céréghino, R. (2013). Mutualistic ants contribute to tank-bromeliad nutrition. *Annals of Botany*, 112 (5), 919-926.

Leroy, C., Carrias, J. F., Céréghino, R., & Corbara, B. (2015). The contribution of microorganisms and metazoans to mineral nutrition in bromeliads. *Journal of Plant Ecology*, 9 (3), 241-255.

Leroy, C., Corbara, B., Dézerald, O., Trzcinski, M. K., Carrias, J. F., Dejean, A., & Céréghino, R. (2017). What drives detrital decomposition in neotropical tank bromeliads?. *Hydrobiologia*, 802 (1), 85-95.

Liria, J. (2007). Fauna fitotelmata en las bromelias *Aechmea fendleri* André y *Hohenbergia stellata* Schult del Parque Nacional San Esteban, Venezuela. *Revista Peruana de Biología*, 14 (1), 33-38.

Looby, C., Hauge, J. B., Barry, D., & Eaton, W. D. (2012). Fungal inhibition by *Bromelia pinguin* (Bromeliaceae) and its effect on nutrient cycle dynamics. *Tropical Ecology*, 53 (2), 225-234.

Mageski, M. M., Ferreira, R. B., Beard, K. H., Costa, L. C., Jesus, P. R., Medeiros, C. C., & Ferreira, P. D. (2016). Bromeliad selection by *Phyllodytes luteolus* (Anura, Hylidae): The influence of plant structure and water quality factors. *Journal of Herpetology*, 50 (1), 108-112.

Marino, N. A., Guariento, R. D., Dib, V., Azevedo, F. D., & Farjalla, V. F. (2011). Habitat size determine algae biomass in tank-bromeliads. *Hydrobiologia*, 678 (1), 191-199.

Marino, N. A., Srivastava, D. S., & Farjalla, V. F. (2016). Predator kairomones change food web structure and function, regardless of cues from consumed prey. *Oikos*, 125 (7), 1017-1026.

Marques, G. R., & Forattini, O. P. (2009). *Anopheles cruzii* larvae found in bromelias in an urban area on the Brazilian coast. *Revista de saude publica*, 43 (2), 369-372.

Marques, T. C., Bourke, B. P., Laporta, G. Z., & Sallum, M. A. M. (2012). *Parasites & vectors*, 16, 5-41.

Marteis, L. S., Natal, D., Sallum, M. A. M., Medeiros-Sousa, A. R., & La Corte, R. (2017). Mosquitoes of the Caatinga: 2. Species from periodic sampling of bromeliads and tree holes in a dry Brazilian forest. *Acta tropica*, 171, 114-123.

Mestre, L. A. M., Aranha, J. M. R., & Esper, M. D. L. P. (2001). Macroinvertebrate fauna associated to the bromeliad *Vriesea inflata* of the Atlantic Forest (Paraná State, Southern Brazil). *Brazilian archives of biology and technology*, 44 (1), 89-94.

Monteiro, R. F., & Macedo, M. V. (2014). First report on the diversity of insects trapped by a sticky exudate of the inflorescences of *Vriesea bituminosa* Wawra (Bromeliaceae: Tillandsioideae). *Arthropod-Plant Interactions*, 8 (6), 519-523.

Montero, G., Feruglio, C., & Barberis, I. M. (2010). The phytotelmata and foliage macrofauna assemblages of a bromeliad species in different habitats and seasons. *Insect Conservation and Diversity*, 3 (2), 92-102.

Morales-Linares, J., García-Franco, J. G., Flores‐Palacios, A., Valenzuela‐González, J. E., Mata-Rosas, M., & Díaz-Castelazo, C. (2017). Spatial structure of ant-gardens: vertical distribution on host trees and succession/segregation of associated vascular epiphytes. *Journal of Vegetation Science*, 28, 1036-1046.

Moura, F. D. S., Vasconcellos, A., de Araujo, V. F. P., & Bandeira, A. G. (2006). Feeding habit of *Constrictotermes cyphergaster* (Isoptera, Termitidae) in an area of Caatinga, Northeast Brazil. *Sociobiology*, 48, 21-26.

Moura, M. R. D., Motta, A. P., & Feio, R. N. (2011). An unusual reproductive mode in Hypsiboas (Anura: Hylidae). *Zoologia* (Curitiba), 28(1), 142-144.

Nesbitt, H. H. J. (1985). A new mite from Bromeliad leaf-axils from Costa Rica (Acari: Acaridae). *International Journal of Acarology*, 11(3), 209-214.

Ngai, J. T., & Srivastava, D. S. (2006). Predators accelerate nutrient cycling in a bromeliad ecosystem. *Science*, 314, 963-963.

Ospina-Bautista, F., Estévez-Varón, J. V., Realpe, E., & Gast, F. (2008). Diversity of aquatic invertebrates associated to Bromeliaceae in the mountain cloud forest. *Revista Colombiana de Entomología*, 34(2), 224-229.

Ospina-Bautista, F., Realpe, E., & Arias-Pineda, J. Y. (2016). Occurrence of *Armases angustum* (Smith, 1870) (Decapoda, Sesarmidae) In tanks of *Guzmania sp.* (Bromeliaceae) at tropical rain forest of Chocó (Colombia). *Boletín Científico. Centro de Museos. Museo de Historia Natural*, 20(2), 173-178.

Osses, F., Martins, E. G., & Machado, G. (2008). Oviposition site selection by the bromeliad-dweller harvestman *Bourguyia hamata* (Arachnida: Opiliones). *Journal of Ethology*, 26 (2), 233-241.

Pansarin, E. R., & de Pedro, S. R. M. (2016). Reproductive biology of a hummingbird‐pollinated Billbergia: light influence on pollinator behaviour and specificity in a Brazilian semi‐deciduous forest. *Plant Biology*, 18 (6), 920-927.

Pederassi, J., Lima, M. S. C. S., Peixoto, O. L., & Souza, C. A. S. (2012). The choice of bromeliads as a microhabitat by *Scinax argyreornatus* (Anura, Hylidae). *Brazilian Journal of Biology*, 72 (2), 229-233.

Pereira, D. L. V., Neiss, U. G., & Ferreira, R. L. M. (2007). Distribution of *Paravelia recens* (Drake & Harris, 1935) (Hemiptera, Heteroptera, Veliidae) in *Guzmania brasiliensis* Ule, 1907 (Bromeliaceae) at Adolpho Ducke Forest Reserve, Amazonas, Brazil. *Acta Amazonica*, 37 (1), 147-150.

Pešić, V., de Oliveira Piccoli, G. C., de Araújo, M. S., & Rezende, J. M. (2015). A new genus of water mites (Acari, Hydrachnidia, Wettinidae) from bromeliad phytotelmata in the Brazilian Atlantic rainforest. *ZooKeys*, 516, 27-33.

Pešić, V., Zawal, A., Piccoli, G. C. D. O., & Gonçalves, A. Z. (2016). New records of water mites (Acari, Hydrachnidia) from bromeliad phytotelmata in Brazilian Atlantic rainforest, with description of one new species. Systematic and Applied *Acarology*, 21 (4), 537-544.

Pett-Ridge, J., & Silver, W. L. (2002). Survival, growth, and ecosystem dynamics of displaced bromeliads in a montane tropical forest. *Biotropica*, 34 (2), 211-224.

Płachno, B. J., Stpiczyńska, M., Davies, K. L., Świątek, P., & de Miranda, V. F. O. (2017). Floral ultrastructure of two Brazilian aquatic-epiphytic bladderworts: *Utricularia cornigera* Studnička and *U. nelumbifolia* Gardner (Lentibulariaceae). *Protoplasma*, 254 (1), 353-366.

Poelman, E. H., & Dicke, M. (2008). Space use of Amazonian poison frogs: testing the reproductive resource defense hypothesis. *Journal of Herpetology*, 42 (2), 270-278.

Poelman, E. H., van Wijngaarden, R. P., & Raaijmakers, C. E. (2013). Amazon poison frogs (*Ranitomeya amazonica*) use different phytotelm characteristics to determine their suitability for egg and tadpole deposition. *Evolutionary ecology*, 27 (4), 661-674.

Pontes, R. C., Santori, R. T., Cunha, F. C., & Pontes, J. A. L. (2013). Habitat selection by anurofauna community at rocky seashore in coastal Atlantic Forest, Southeastern Brazil. *Brazilian Journal of Biology*, 73 (3), 533-542

Richardson, B. A., Richardson, M. J., Scatena, F. N., & McDowell, W. H. (2000a). Effects of Nutrient Availability and Other Elevational Changes on Bromeliad Populations and Their Invertebrate Communities in a Humid Tropical Forest in Puerto Rico. *Journal of Tropical Ecology,* 16 (2), 167-188.

Richardson, B. A., Rogers, C., & Richardson, M. J. (2000b). Nutrients, diversity, and community structure of two phytotelm systems in a lower montane forest, Puerto Rico. *Ecological Entomology*, 25 (3), 348-356.

Ríos, L. D., & Cascante-Marín, A. (2017). High selfing capability and low pollinator visitation in the hummingbird-pollinated epiphyte *Pitcairnia heterophylla* (Bromeliaceae) at a Costa Rican mountain forest. *Revista de Biología Tropical*, 65 (2), 735-743.

Robbins, R, K. (2010). The "upside down" systematics of hairstreak butterflies (Lycaenidae) that eat pineapple and other Bromeliaceae. *Studues on neotropical Fauna and Enviroment*, 45 (1), 21-37.

Robbins, R. K., & Nicolay, S. S. (2001). An overview of Strymon Hübner (Lycaenidae: Theclinae: Eumaeini). *Journal of the Lepidopterists' Society,* 55, 85–100.

Rocca, M. A., & Sazima, M. (2013). Quantity versus quality: identifying the most effective pollinators of the hummingbird-pollinated *Vriesea rodigasiana* (Bromeliaceae). *Plant systematics and evolution*, 299 (1), 97-105.

Romero, G.Q., Mazzafera, P., Vasconcellos-Neto, J., & Trivelin, P.C.O. (2006) Bromeliad-living spiders improve host plant nutrition and growth. *Ecology*, 87, 803-808.

Romero, G. Q., Vasconcellos-Neto, J., & Trivelin, P. C. (2008). Spatial variation in the strength of mutualism between a jumping spider and a terrestrial bromeliad: evidence from the stable isotope 15N. *Acta Oecologica*, 33 (3), 380-386.

Romero, G. Q., Nomura, F., Gonçalves, A. Z., Dias, N. Y., Mercier, H., Conforto, E. D. C., & Rossa-Feres, D. D. C. (2010). Nitrogen fluxes from treefrogs to tank epiphytic bromeliads: an isotopic and physiological approach. *Oecologia*, 162 (4), 941-949.

Rosati, V. R., & Bucher, E. H. (1992). Seasonal diet of the Chacoan Cavy (*Pediolagus salinicola*) in the western Chaco, *Argentina. Mammalia*, 56 (4), 567-574.

Rotheray, G. E., Hancock, E. G., & Marcos-García, M. A. (2007). Neotropical Copestylum (Diptera, Syrphidae) breeding in bromeliads (Bromeliaceae) including 22 new species. *Zoological Journal of the Linnean Society*, 150 (2), 267-317.

Ruano-Fajardo, G., Rovito, S. M., & Ladle, R. J. (2014). Bromeliad selection by two salamander species in a harsh environment. *PloS one*, 9 (6), e98474.

Sabagh, L. T., Ferreira, G. L., Branco, C. W., Rocha, C. F. D., & Dias, N. Y. (2012). Larval diet in bromeliad pools: a case study of tadpoles of two species in the genus Scinax (Hylidae). *Copeia*, 4, 683-689.

Santana, C. S., & Machado, C. G. (2010). Fenologia de floração e polinização de espécies ornitófilas de bromeliáceas em uma área de campo rupestre da Chapada Diamantina, BA, Brasil. *Brazilian Journal of Botany*, 33(3), 469-477.

Sazima, I., Vogel, S., & Sazima, M. (1989). Bat pollination of *Encholirium glaziovii*, a terrestrial bromeliad. *Plant Systematics and Evolution*, 168 (3-4), 167-179.

Sazima, I., Buzato, S., & Sazima, M. (1995). The Saw-billed Hermit *Ramphodon naevius* and its flowers in southeastern Brazil. *Journal für ornithologie*, 136 (2), 195-206.

Sazima, I., Buzato, S., & Sazima, M. (1996). An assemblage of hummingbird‐pollinated flowers in a montane forest in southeastern Brazil. *Botanica Acta*, 109 (2), 149-160.

Sazima, M., & Sazima, I. (1999). The perching bird *Coereba flaveola* as a co-pollinator of bromeliad flowers in southeastern Brazil. *Canadian journal of zoology*, 77 (1), 47-51.

Sazima, M., Buzato, S., & Sazima, I. (1999). Bat-pollinated flower assemblages and bat visitors at two Atlantic forest sites in Brazil. *Annals of Botany*, 83 (6), 705-712.

Schmelz, R. M., Jocque, M., & Collado, R. (2015). Microdrile Oligochaeta in bromeliad pools of a Honduran cloud forest. *Zootaxa*, 3947 (4), 508-526.

Schmid, S., Schmid, V. S., Zillikens, A., & Steiner, J. (2011a). Diversity of flower visitors and their role for pollination in the ornithophilous bromeliad *Vriesea friburgensis* in two different habitats in southern Brazil. *Ecotropica*, 17(1), 91-102.

Schmid, S., Schmid, V. S., Zillikens, A., Harter‐Marques, B., & Steiner, J. (2011b). Bimodal pollination system of the bromeliad *Aechmea nudicaulis* involving hummingbirds and bees. *Plant Biology*, 13, 41-50.

Schmidt, G., & Zotz, G. (2000). Herbivory in the epiphyte, *Vriesea sanguinolenta* Cogn. & Marchal (Bromeliaceae). *Journal of Tropical Ecology*, 16 (6), 829-839.

Silva, H. R. D., Carvalho, A. L. G. D., & Bittencourt‐Silva, G. B. (2011). Selecting a hiding place: anuran diversity and the use of bromeliads in a threatened coastal sand dune habitat in Brazil. *Biotropica*, 43 (2), 218-227.

Siqueira Filho, J. A. D., & Machado, I. C. S. (2001). Reproductive biology of *Canistrum aurantiacum* e morren (Bromeliaceae) in atlantic rain forest northeastern Brazil. *Acta Botanica Brasilica*, 15 (3), 427-443.

Smirnov, N. N. (1988). Cladocera (Crustacea) from Nicaragua. *Hydrobiologia*, 160(1), 63-77.

Sodré, V. M., Rocha, O., & Messias, M. C. (2010). Chironomid larvae inhabiting bromeliad phytotelmata in a fragment of the Atlantic Rainforest in Rio de Janeiro State. *Brazilian Journal of Biology*, 70 (3), 587-592.

Souza, F. L., Uetanabaro, M., Landgref Filho, P., & Faggioni, G. (2009). An alternative water source for the Blaze-winged Parakeet, *Pyrrhura devillei*?.*Revista Brasileira de Ornitologia-Brazilian Journal of Ornithology*, 17, 210-212.

Souza, R. C. D., Pereira, M. G., Menezes, L. F. T. D., Silveira Filho, T. B., & Silva, A. N. D. (2016). Role of terrestrial bromeliads in nutrient cycling, Restinga da Marambaia, Brazil. *Floresta e Ambiente*, 23 (2), 161-169.

Srivastava, D. S., Melnychuk, M. C., & Ngai, J. T. (2005). Landscape variation in the larval density of a bromeliad-dwelling zygopteran, *Mecistogaster modesta*(Odonata: Pseudostigmatidae). International *Journal of Odonatology*, 8(1), 67-79.

Starzomski, B. M., Suen, D., & Srivastava, D. S. (2010). Predation and facilitation determine chironomid emergence in a bromeliad‐insect food web. *Ecological Entomology*, 35 (1), 53-60.

Suárez, L. (1988). Seasonal distribution and food habits of spectacled bears *Tremarctos ornatus* in the highlands of Ecuador. *Studies on neotropical fauna and environment*, 23 (3), 133-136.

Suleiman, M., Brandt, F. B., Brenzinger, K., Martinson, G. O., & Braker, G. (2017). Potential N 2 O Emissions from the Tanks of Bromeliads Suggest an Additional Source of N 2 O in the Neotropics. *Microbial ecology*, 73 (4), 751-754.

Talaga, S., Dézerald, O., Carteron, A., Petitclerc, F., Leroy, C., Céréghino, R., & Dejean, A. (2015). Tank bromeliads as natural microcosms: a facultative association with ants influences the aquatic invertebrate community structure. *Comptes Rendus Biologies*, 338 (10), 696-700.

Teixeira, R. L., Schineider, J. A. P., & Almeida, G. I. (2002). The occurrence of amphibians in bromeliads from a southeastern Brazilian restinga habitat, with special reference to *Aparasphenodon brunoi* (Anura, Hylidae). *Brazilian Journal of Biology*, 62 (2), 263-268.

Teixeira, R. L., Zamprogno, C., Almeida, G. I., & Schineider, J. A. P. (1997). Ecological topics of *Phyllodytes luteolus*(Amphibia, Hylidae) in a sandy coastal plain of Sao Mateus, Espirito Santo State. *Revista Brasileira de Biologia*, 57 (4), 647-654.

Ticktin, T. (2003). Relationships between El Niño Southern Oscillation and demographic patterns in a substitute food for collared peccaries in Panama. *Biotropica*, 35 (2), 189-197.

Torreias, S. R. D. S., Ferreira-Keppler, R. L., Godoy, B. S., & Hamada, N. (2010). Mosquitoes (Diptera, Culicidae) inhabiting foliar tanks of *Guzmania brasiliensis* Ule (Bromeliaceae) in central Amazonia, Brazil. *Revista Brasileira de Entomologia*, 54 (4), 618-623.

Torreias, S. R. D. S., Neiss, U. G., Hamada, N., Ferreira-Keppler, R. L., & Lencioni, F. A. (2008). Description of the larva of *Bromeliagrion rehni* (Odonata: Coenagrionidae) with bionomic notes concerning its phytotelmic habitat in central Amazonas, Brazil. *Revista Brasileira de Zoologia*, 25 (3), 479-486.

Torreias, S. R. D. S., & Ferreira-Keppler, R. L. (2011). Macroinvertebrates inhabiting the tank leaf terrestrial and epiphyte bromeliads at Reserva Adolpho Ducke, Manaus, Amazonas.

*Brazilian Archives of Biology and Technology*, 54 (6), 1193-1202.

Torresdal, J. D., Farrell, A. D., & Goldberg, C. S. (2017). Environmental DNA detection of the golden tree frog (*Phytotriades auratus*) in bromeliads. *PloS one*, 12 (1), e0168787.

Tschapka, M., & Von Helversen, O. (2007). Phenology, nectar production and visitation behaviour of bats on the flowers of the bromeliad *Werauhia gladioliflora* in a Costa Rican lowland rain forest. *Journal of Tropical Ecology*, 23 (4), 385-395.

Vásquez-Almazán, C. R., Rovito, S. M., Good, D. A., & Wake, D. B. (2009). A new species of Cryptotriton (Caudata: Plethodontidae) from eastern Guatemala. *Copeia*, 2, 313-319.

Vrcibradic, D., & Rocha, C. F. D. (1996). Ecological differences in tropical sympatric skinks (*Mabuya macrorhyncha* and *Mabuya agilis*) in southeastern Brazil. *Journal of Herpetology*, 30, 60-67.

Wagner, R., Richardson, B. A., & Richardson, M. J. (2010). A new psychodid species from Saban tank bromeliads. *Studies on Neotropical Fauna and Environment*, 45 (3), 121-127.

Wehrtmann, I. S., Magalhães, C., & Bello-González, O. C. (2016). First confirmed report of a primary freshwater crab (Brachyura: Pseudothelphusidae) associated with bromeliads in the Neotropics. *Journal of Crustacean Biology*, 36 (3), 303-309.

Wendt, T., Canela, M. B. F., Gelli de Faria, A. P., & Rios, R. I. (2001). Reproductive biology and natural hybridization between two endemic species of *Pitcairnia* (Bromeliaceae). *American journal of Botany*, 88 (10), 1760-1767.

Weygoldt, P. (1989). Feeding behavior of the larvae of *Fritziana goeldi*(Anura, Hylidae). *Amphibia-Reptilia*, 10 (4), 419-422.

Wittman, P. K. (2000). The animal community associated with canopy bromeliads of the lowland Peruvian Amazon rainforest. *Selbyana*, 21(1.2), 48-51.

Zillikens, A., Steiner, J., & Mihalkó, Z. (2001). Nests of *Augochlora* (A.) *esox* in bromeliads, a previously unknown site for sweat bees (Hymenoptera: Halictidae). *Studies on Neotropical Fauna and Environment*, 36(2), 137-142.
