# Supplementary material for: Ecosystem services provided by bromeliad plants: A systematic review
Source: Ecol Evol. 2019 May 29;9(12):7360–72. doi: 10.1002/ece3.5296 (PMC6662323; doi:10.1002/ece3.5296)
Supplement: Supplementary file 3 [file ECE3-9-7360-s003.docx]

**APPENDIX 3.** Provisioning services.

**Appendix 3A.** Chemical products extracted from bromeliads and their uses by human society.

| **Species** | **Chemical products** | **Uses** | **Reference** |
| --- | --- | --- | --- |
| *Ananas comosus* | Proteases, alkaloids, flavonoids, phenols, tannins, steroids | Meat industry, nutritional supplement | Kalaiselvi *et al*., 2012; Rowan *et al*., 1990. |
| *Ananas sp.* | Terpenes, flavonoids, steroids, hydroxycinnamic acids, phenylpropane diglycerides, lignans | Food industry, perfumery, cosmetics, therapeutic and antibacterial uses | Hilo de Souza *et al*., 2016; Manetti *et al*., 2009. |
| *Aechmea sp.* | Terpenoids, volatile compounds | Food industry, perfumery and cosmetics | Hilo de Souza *et al*., 2016. |
| *Bromelia antiacantha* | Flavones, hydroxycinnamic acids, saponins, phenolic compounds, bioactive components* | Nutritional supplement, antioxidant, cytotoxic activity | Krumreich *et al*., 2015; Manetti *et al*., 2010; Santos *et al*., 2008. |
| *Bromelia balansae* | Proteases | Milk coagulation | Pardo *et al*., 2001. |
| *Bromelia hemisphaerica* | Fungicides | Inhibition of sporulation of *Rhizopus sp.* | Bautista-Baños *et al*., 2000. |
| *Bromelia karatas* | Flavonoids | Nutritional supplement | Osorio *et al*., 2016. |
| *Bromelia laciniosa* | Flavonoids, carbohydrates, non-polar natural products | Antibacterial, nutritional supplement | Chaves *et al*., 2015; Da Silva *et al*., 2014; Juvik *et al*., 2017. |
| *Bromelia pinguin* | Tannins, flavonoids | Antibacterial | Pío-Léon *et al*., 2009. |
| *Bromelia sp.* | Terpenes, flavonoids, steroids, hydroxycinnamic acids, phenylpropane diglycerides, lignans | Therapeutic and antibacterial uses | Manetti *et al*., 2009. |
| *Encholirium spectabile* | Flavonoids, non-polar natural products | Antibacterial, nutritional supplement | Fernandes *et al*., 2015; Juvik *et al*., 2017. |
| *Neoglaziovia variegata* | Flavonoids, bioacaricide, non-polar natural products | Antibacterial, biologic control, nutritional supplement | Dantas *et al*., 2015; Fernandes *et al*., 2015; Juvik *et al*., 2017. |
| *Nidullarium procerum* | Terpenes, flavonoids, steroids, hydroxycinnamic acids, phenylpropane diglycerides, lignans | Therapeutic and antibacterial uses | Manetti *et al*., 2009. |
| *Pseudananas macrodontes* | Macrodontin | Proteolytic food | Natalucci *et al*., 1995. |
| *Tillandsia sp.* | Terpenes, flavonoids, steroids, hydroxycinnamic acids, phenylpropane diglycerides, lignans | Therapeutic and antibacterial uses | Manetti *et al*., 2009. |
| *Vriesea sp.* | Terpenoids, volatile compounds | Food industry, perfumery and cosmetics | Hilo de Souza *et al*., 2016. |

* Carbohydrates, lipids, palmitic acid, linoleic acid and oleic acid.

**Appendix 3B.** Pharmaceutical use of bromeliad species.

| **Bromeliad species** | **Pharmaceutical potential** | **Reference** |
| --- | --- | --- |
| *Ananas comosus* | Anti-inflammatory, nutraceutical, chemopreventive, antiproliferative, proapoptotic, antirheumatic | Darshan & Doreswamy, 2004; Milić *et al*., 2014; Kargutkar & Brijesh, 2016; Riya *et al*., 2014; Romano *et al*., 2014 |
| *Ananas ananassoides* | Antiulcerogenic | Silva *et al*., 2008 |
| *Bromelia plumieri* | Hypoglycemic | Andrade-Cetto & Medina Hernandez, 2013 |
| *Bromelia sp.* | Anti-inflammatory | Darshan & Doreswamy, 2004 |
| *Encholirium spectabile* | Antiulcer | Moraes de Carvalho *et al*., 2010 |
| *Nidularium procerum* | Anti-inflammatory, antiallergic | Amendoeira *et al*., 2005; Vieira-de-Abreu *et al*., 2005 |
| *Pseudananas macrodontes* | Anti-inflammatory | Errasti *et al*., 2013 |
| *Tillandsia recurvata* | Antitumor, anti-inflammatory | De Vasconcelos *et al*., 2013 |

**REFERENCES**

Amendoeira, F.C., Frutuoso, V.D., Zanon, C., Chedier, L.M., Figueiredo, M.R., Kaplan, M.A., Bandeira-Melo, C., Bozza, P.T., & Castro-Faria-Neto, H.C. (2005). Anti-inflammatory activity in the aqueous crude extract of the leaves of *Nidularium procerum*: A bromeliaceae from the Brazilian coastal rain forest. *Biological and pharmaceutical bulletin, 28(6)*, 1010-1015.

Andrade-Cetto, A. D., & Medina-Hernández, A. E. B. (2013). Hypoglycemic effect of *Bromelia plumieri* (E. Morren) LB Sm., leaves in STZ-NA-induced diabetic rats. *Frontiers in pharmacology*, 36, 1-4.

Bautista-Baños, S., Hernández-López, M., Dıaz-Pérez, J. C., & Cano-Ochoa, C. F. (2000). Evaluation of the fungicidal properties of plant extracts to reduce *Rhizopus stolonifer* of ‘ciruela’fruit (*Spondias purpurea* L.) during storage. *Postharvest Biology and Technology*, 20(1), 99-106.

Chaves, E. M. F., Silva, J. N., Alessandro, L. I. M. A., Albuquerque, U. P., & Barros, R. F. M. (2015). Potential of wild food plants from the semi-arid region of northeast Brazil: chemical approach ethnoguided. *Espacios*, 36 (16), 20.

Da Silva, V. F., Franco, I., Damasceno, T. E. F., Da Silva Almeida, J. R. G., & da Costa, M. M. (2014). Antimicrobial potential of ethanol extracts of plants against gram-negative bacilli isolated from cervicovaginal mucosa of sheep bred in the region of Petrolina-PE. *Semina: Ciências Agrárias*, 35(2), 883-890.

Dantas, A. C. S., Machado, D. M. R., Araujo, A. C., Oliveira-Junior, R. G., Lima-Saraiva, S. R. G., Ribeiro, L. A. A., Almeida, J.R.G.S., & Horta, M. C. (2015). Acaricidal activity of extracts from the leaves and aerial parts of *Neoglaziovia variegata* (Bromeliaceae) on the cattle tick Rhipicephalus (Boophilus) microplus. *Research in veterinary science*, 100, 165-168.

Darshan, S., & Doreswamy, R. (2004). Patented antiinﬂammatory plant drug development from traditional medicine. *Phytotherapy Research*, 18(5), 343-357.

De Vasconcelos, A. L., Ximenes, E. A., Soares, L. A. L., & Randau, K. P. (2013). *Tillandsia recurvata* L. (Bromeliaceae): A pharmacognostic study. *Revista de Ciencias Farmaceuticas Basica e Aplicada*, 34(2),151-159.

Errasti, M. E., Caffini, N. O., Pelzer, L. E., & Rotelli, A. E. (2013). Evaluation of anti-inflammatory activity of *Pseudananas macrodontes* (Morr.) Harms (Bromeliaceae) fruit extract in rats. *Zeitschrift für Naturforschung C*, 68(11-12), 445-452.

Fernandes, A., Aquino, S., Gouveia, G., Almeida, J., & Costa, M. (2015). Antimicrobial activity in vitro extracts from caatinga plants in isolated *Escherichia coli* from pigs. *Revista Brasileira de Plantas Medicinais*, 17(4), 1097-1102.

Hilo de Souza, E., Massarioli, A. P., Moreno, I. A., Souza, F. V., Ledo, C. A., Alencar, S. M., & Martinelli, A. P. (2016). Volatile compounds profile of Bromeliaceae flowers. *Revista de Biología Tropical,* 64(3), 1101-1116.

Juvik, O. J., Holmelid, B., Francis, G. W., Lie Andersen, H., de Oliveira, A. P., Gonçalves de Oliveira Júnior, R., Gonçalves de Oliveira, R., Guedes da Silva, J., & Fossen, T. (2017). Non-Polar Natural Products from *Bromelia laciniosa*, *Neoglaziovia variegata*, and *Encholirium spectabile*(Bromeliaceae). *Molecules*, 22(9), 1478.

Kalaiselvi, M., Gomathi, D., & Uma, C. (2012). Occurrence of Bioactive compounds in *Ananus comosus* (L.): A quality Standardization by HPTLC. *Asian Pacific Journal of Tropical Biomedicine*, 2(3), S1341-S1346.

Kargutkar, S., & Brijesh, S. (2016). Anti-rheumatic activity of *Ananas comosus* fruit peel extract in a complete Freund’s adjuvant rat model. *Pharmaceutical biology*, 54(11), 2616-2622.

Krumreich, F. D., Corrêa, A. P. A., Silva, S. D. S. D., & Zambiazi, R. C. (2015). Physical and chemical composition and bioactive compounds in *Bromelia antiacantha* bertol. Fruits. *Revista Brasileira de Fruticultura*, 37(2), 450-456.

Manetti, L. M., Delaporte, R. H., & Laverde Jr, A. (2009). Secondary metabolites from Bromeliaceae family. *Química Nova*, 32(7), 1885-1897.

Manetti, L. M., Turra, A. F., Takemura, O. S., Svidzinski, T. I. E., & Laverde Junior, A. (2010). Evaluation of antimicrobial, cytotoxic, molluscicidal and antioxidant activities of *Bromelia antiacantha* Bertol.(Bromeliaceae). *Revista Brasileira de Plantas Medicinais*, 12(4), 406-413.

Milić, N., Milanović, M., Goločorbin-Kon, S., & Milošević, N. (2014). Current experience and future perspectives of bromelain application in medicine.*Medicinski Casopis*, 48(4), 153-158.

Moraes de Carvalho, K. I., Fernandes, H. B., Frota Machado, F. D., Oliveira, I. S., Oliveira, F. A., Nunes, P. H. M, Lima, J.T., Almeida, J.R.,& Oliveira, R. C. (2010). Antiulcer activity of ethanolic extract of *Encholirium spectabile* Mart. ex Schult & Schult f. (Bromeliaceae) in rodents. *Biological Research*, 43(4), 459-465.

Natalucci, C. L., Brullo, A., López, L. M., Hilal, R. M., & Caffini, N. O. (1995). Macrodontin, a new protease isolated from fruits of *Pseudananas macrodontes* (Morr.) Harms (Bromeliaceae). *Journal of Food Biochemistry*, 19(6), 443-454.

Osorio, M. N., Moyano, D. F., Murillo, W., Murillo, E., Ibarz, A., & Solanilla, J. F. (2016). Functional and rheological properties of piñuela (*Bromelia karatas*) in two ripening stages.*International Journal of Food Engineering*, 13(1), 13.

Pardo, M. F., López, L. M., Caffini, N. O., & Natalucci, C. L. (2001). Properties of a milk clotting protease isolated from fruits of *Bromelia balansae* Mez. *Biological Chemistry,* 382(5), 871-874.

Pío-León, J. F., López-Angulo, G., Paredes-López, O., de Jesús Uribe-Beltrán, M., Díaz-Camacho, S. P., & Delgado-Vargas, F. (2009). Physicochemical, nutritional and antibacterial characteristics of the fruit of *Bromelia pinguin* L. *Plant Foods for Human Nutrition*, 64(3), 181-187.

Riya, M. P., Antu, K. A., Vinu, T., Chandrakanth, K. C., Anilkumar, K. S., & Raghu, K. G. (2014). An in vitro study reveals nutraceutical properties of *Ananas comosus* (L.) Merr. var. Mauritius fruit residue beneficial to diabetes. *Journal of the Science of Food and Agriculture*, 94(5), 943-950.

Romano, B., Fasolino, I., Pagano, E., Capasso, R., Pace, S., De Rosa, G., Millic, N., Orlando, P., Izzo, A.A., & Borrelli, F. (2014). The chemopreventive action of bromelain, from pineapple stem (*Ananas comosus* L.), on colon carcinogenesis is related to antiproliferative and proapoptotic effects.*Molecular Nutrition & Food Research*, 58(3), 457-465.

Rowan, A. D., Buttle, D. J., & Barrett, A. J. (1990). The cysteine proteinases of the pineapple plant. *Biochemical Journal*, 266(3), 869-875.

Santos, V. N., Freitas, R. A. D., Deschs, F. C., & Biavatti, M. W. (2008). Ripe fruits of Bromelia antiacantha: investigations on the chemical and bioactivity profile. *Revista Brasileira de Farmacognosia*, 19, 358-365.

Silva, J. S., Andreo, M. A., Tubaldini, F. R., Varanda, E. A., Rocha, L. R. M., Brito, A. R. M. S., Vilegas, W., & Hiruma-Lima, C. A. (2008). Differences in gastroprotective and mutagenic actions between polar and apolar extracts of *Ananas ananassoides*. *Journal of Medicinal Food*, 11(1), 160-168.

Vieira-de-Abreu, A., Amendoeira, F. C., Gomes, G. S., Zanon, C., Chedier, L. M., Figueiredo, M. R., Kaplan, M.A., Frutuoso, V.S., Castro-Faria-Neto, H.C., Weller, P.F., Bandeira-Melo, C., & Bozza, P.T. (2005). Anti-allergic properties of the bromeliaceae *Nidularium procerum*: inhibition of eosinophil activation and influx. *International Immunopharmacology*, 5(13-14), 1966-1974.
