# Supplementary material for: Ecosystem services provided by bromeliad plants: A systematic review
Source: Ecol Evol. 2019 May 29;9(12):7360–72. doi: 10.1002/ece3.5296 (PMC6662323; doi:10.1002/ece3.5296)
Supplement: Supplementary file 4 [file ECE3-9-7360-s004.docx]

**APPENDIX 4. Regulating services provided by bromeliads.**

**Appendix 4A.** References for classification of regulating services provided by bromeliads.

| **Services** | **References** |
| --- | --- |
| Diseases regulation | Blooi *et al*., 2017; Burrowes *et al*., 2017; Hammill *et al.*2015; Lounibos *et al*., 2003; O Meara *et al*., 2003; Santos *et al*., 2011. |
| Water regulation | Caballero-Rueda *et al*., 1997; Cogliatti-Carvalho *et al*., 2010; Fish, 1983; Guevara-Escobar *et al.,*2011; Guimaraes-Souza *et al*., 2006; Martin & Schmitt, 1989; Martorell & Ezcurra, 2007; Van Stan & Pypker, 2015; Zotz & Vera, 1999. |
| CO_2_ and methane capture | Atwood *et al*., 2013; Brandt *et al.*, 2017; Caballero-Rueda *et al*., 1997; Goffredi *et al*., 2011; Martinson *et al.*, 2010; Oliveira, 2004; Pierce *et al*., 2002; Richardson *et al*., 2000 a, b. |

**Appendix 4B.** Water amount reserved by tank bromeliad species according with the habitat. nr: not recorded.

| **Country** | **Habitat** | **Elevation (m)** | **Bromeliad species** | **Number of bromeliads** | **Water amount (ml)**  $\bar{\boldsymbol{x}}$ ± **sd** | **Reference** |
| --- | --- | --- | --- | --- | --- | --- |
| Brazil | Closed "restinga" vegetation. | 9 | *Quesnelia* | 20 | 360 ± nr | Farjalla *et al.*, 2016. |
| Brazil | Open "restinga" vegetation. | 9 | *Quesnelia* | 20 | 910 ± nr | Farjalla *et al.*, 2016. |
| Brazil | Open "restinga" vegetation | 9 | *Neoregelia* | 26 | 300 ± nr | Farjalla *et al.*, 2016. |
| Brazil | Open bushy of *Clusia hilariana* | nr | *Negeria cruenta* | 8 | 392.8 ± 91.3 | Guimaraes-Souiza *et al.,* 2006. |
| Brazil | Open bushy of *Clusia hilariana* | nr | *Aechmea nudicaulis* | 8 | 179.7 ± 65.7 | Guimaraes-Souiza *et al.,* 2006. |
| Brazil | "restinga" vegetation | nr | *Aechmea nudicaulis* | 16 | 347 ± 160 | Marino *et al.*, 2012. |
| Brazil | "restinga" vegetation | nr | *Neoregelia cruenta* | 16 | 940 ± 429 | Marino *et al.*, 2012. |
| Brazil | "restinga" vegetation | nr | *Vriesea neoglutinosa* | 16 | 1483 ± 723 | Marino *et al.*, 2012. |
| Brazil | "restinga" vegetation | nr | *Aechmea lingulata* | 16 | 1184 ± 625 | Marino *et al.*, 2012. |
| Colombia | Montane cloud forest | 3000 | *Tillandsia turneri* | 37 | 348.5 ± 42.5 | Ospina-Bautista *et al.*, 2004. |
| Colombia | Montane cloud forest | 3100 | *Tillandsia turneri* | 17 | 204 ± 25.88 | Ospina- Bautista *et al.*, 2008. |
| Colombia | Montane cloud forest | 3100 | *Tillandsia complanata* | 17 | 177 ± 51.25 | Ospina- Bautista *et al.*, 2008. |
| French Guiana | Primary rain forest | 80-130 | *Guzmania lingulata* | 38 | 16.1 ± 9.6 | Brouard *et al.*, 2012. |
| French Guiana | Transitional forest | 80-130 | *Guzmania lingulata* | 17 | 16.1 ± 9.6 | Brouard *et al.*, 2012. |
| French Guiana | Transitional forest | 130 | *Aechmea melinonii* | 30 | 134 ± 105.2 | Brouard *et al.*, 2012. |
| French Guiana | Transitional forest | 130 | *Vriesea pleiosticha* | 32 | 71.7 ± 49.8 | Brouard *et al.*, 2012. |
| French Guiana | Inselberg open area | 390 | *Vriesea splendens* | 31 | 50.8 ± 27.8 | Brouard *et al.*, 2012. |
| French Guiana | Inselberg forest area | 160 | *Pitcairnia geyskesii* | 37 | 8.3 ± 1.6 | Brouard *et al.*, 2012. |
| French Guiana | Summit inselberg open área | 420 | *Catopsis berteroniana* | 32 | 40.6 ± 20.2 | Brouard *et al.*, 2012. |
| French Guiana | Rock savannah | nr | Catopsis berteroniana | 29 | 40.6 ± 3.7 | Dézerald *et al.*, 2014. |
| French Guiana | Rock savannah | nr | Aechmea aquilega | 31 | 949.2 ± 102.6 | Dézerald *et al.*, 2014. |
| French Guiana | Transition forest | 130 | *Vriesea pleiosticha* | 30 | 73.2 ± 9.11 | Dézerald *et al.*, 2014. |
| French Guiana | Transition forest | 130 | *Aechmea bromeliifolia* | 26 | 137.8 ± 21.1 | Dézerald *et al.*, 2014. |
| French Guiana | Primary rain forest | 80-130 | *Vriesea splendens* | 26 | 48.5 ± 5.0 | Dézerald *et al.*, 2014. |
| French Guiana | Primary rain forest | 80-130 | Guzmania lingulata | 19 | 17.5 ± 2.5 | Dézerald *et al.*, 2014. |
| French Guiana | Pioneer growth |  | Aechmea mertensii | 66 | 84.4 ± 10.1 | Dézerald *et al.*, 2014. |
| French Guiana | Primary rain forest | 80-130 | Vriesea splendens | 34 | 26.2 ± 3.8 | Dézerald *et al.*, 2014. |
| French Guiana | Pioneer growth | nr | Aechmea mertensii | 45 | 92.4 ± 11.8 | Dézerald *et al.*, 2014. |
| French Guiana | Citrus plantation | nr | Aechmea mertensii | 35 | 31.0 ± 4.5 | Dézerald *et al.*, 2014. |
| French Guiana | Citrus plantation | nr | Aechmea mertensii | 27 | 56.4 ± 8.3 | Dézerald *et al.*, 2014. |
| French Guiana | Primary forest | 80 | *Guzmania, Vriesia, Aechmea* | 20 | 180 ± nr | Farjalla *et al.*, 2016 |
| French Guiana | Forest edge | 80 | *Guzmania, Vriesia, Aechmea* | 20 | 680 ± nr | Farjalla *et al.*, 2016. |
| Puerto Rico | Montane rain forest | 750-815 | *Guzmania berteroniana, Guzmania lingulata, Vriesea sintenissi* | 20 | 134± nr | Richardson *et al.*, 2000. |
| Puerto Rico | Dwarf forest | 950- 980 | *Guzmania berteroniana, Guzmania lingulata, Vriesea sintenissi* | 20 | 103 ± nr | Richardson *et al.*, 2000. |
| Puerto Rico | Secondary subtropical wet forest | 350-400 | *Guzmania* | 10 | 80 ± nr | Farjalla *et al.*, 2016. |
| Puerto Rico | Montane rain forest | 29-405 | *Guzmania berteroniana, Guzmania lingulata, Vriesea sintenissi* | 20 | 166 ± nr | Richardson *et al.*, 2000. |

**REFERENCES**

Atwood, T. B., Hammill, E., Greig, H. S., Kratina, P., Shurin, J. B., Srivastava, D. S., & Richardson, J. S. (2013). Predator-induced reduction of freshwater carbon dioxide emissions. *Nature Geoscience*, 6(3), 191-194.

Blooi, M., Laking, A. E., Martel, A., Haesebrouck, F., Jocque, M., Brown, T., Green, S., Vences, M., Bletz, M., & Pasmans, F. (2017). Host niche may determine disease-driven extinction risk. *PloS one*, 12(7), e0181051.

Brandt, F. B., Martinson, G. O., & Conrad, R. (2017). Bromeliad tanks are unique habitats for microbial communities involved in methane turnover. *Plant and Soil*, 410 (1-2), 167-179.

Brouard, O., Cereghino, R., Corbara, B., Leroy, C., Pelozuelo, L., Dejean, A., & Carrias, J. F. (2012). Understorey environments influence functional diversity in tank‐bromeliad ecosystems. *Freshwater Biology*, 57(4), 815-823.

Burrowes, P. A., Martes, M. C., Torres-Ríos, M., & Longo, A. V. (2017). Arboreality predicts *Batrachochytrium dendrobatidis* infection level in tropical direct-developing frogs. *Journal of Natural History*, 51(11-12), 643-656.

Caballero-Rueda, L. M., Rodriguez, N. & Martin, C. (1997). Dynamics of elements in epiphytes of a high Andean forest in the eastern range of Colombia. *Caldasia*, 19(1-2), 311-322.

Cogliatti-Carvalho, L., Rocha-Pessôa, T. C., Nunes-Freitas, A. F., & Rocha, C. F. D. (2010). Water volume stored in bromeliad tanks in Brazilian restinga habitats. *Acta Botanica Brasilica*, 24(1), 84-95.

Dézerald, O., Talaga, S., Leroy, C., Carrias, J. F., Corbara, B., Dejean, A., & Céréghino, R. (2014). Environmental determinants of macroinvertebrate diversity in small water bodies: insights from tank-bromeliads. *Hydrobiologia*, 723(1), 77-86.

Farjalla, V. F., González, A. L., Céréghino, R. , Dézerald, O. , Marino, N. A., Piccoli, G. C., Richardson, B. A., Richardson, M. J., Romero, G. Q., & Srivastava, D. S. (2016). Terrestrial support of aquatic food webs depends on light inputs: a geographically‐replicated test using tank bromeliads. *Ecology*, 97, 2147-2156.

Fish, D. (1983). Phytotelmata: Flora and Fauna in Frank, J.H. and Lounibos, L.P., (eds.) *Phytotelmata: terrestrial plants as hosts for aquatic insect communities.* Plexus publishing, New Jersey. pp.1-25.

Goffredi, S. K., Jang, G., Woodside, W. T., & Ussler, W. (2011). Bromeliad catchments as habitats for methanogenesis in tropical rainforest canopies. *Frontiers in Microbiology*, 2, 256.

Guevara-Escobar, A., Cervantes-Jiménez, M., Suza´n-Azpiri, H., González-Sosa, E., Hernández-Sandoval, L., Malda-Barrera, G., & Martínez-Díaz, M. (2011). Fog interception by Ball moss (*Tillandsia recurvata*). *Hydrology and Earth System Sciences*, 15, 2509-2518.

Guimaraes-Souza, B. A., Mendes, G. B., Bento, L., Marotta, H., Santoro, A. L., Esteves, F. A., Pinho, L., Farjalla, V.F., & Enrich-Prast, A. (2006). Limnological parameters in the water accumulated in tropical bromeliads. *Acta Limnologica Brasiliensia*, 18(1), 47-53.

Hammill, E., Atwood, T., & Srivastava, S.D. (2015). Predation Threat Alters Composition and Functioning of Bromeliad Ecosystems. *Ecosystems,* 18, 857-866.

Lounibos, L. P., O'meara, G. F., Nishimura, N., & Escher, R. L. (2003). Interactions with native mosquito larvae regulate the production of *Aedes albopictus* from bromeliads in Florida. *Ecological Entomology*, 28(5), 551-558.

Marino, N. A., Srivastava, D. S., & Farjalla, V. F. (2013), Aquatic macroinvertebrate community composition in tank‐bromeliads is determined by bromeliad species and its constrained characteristics. *Insect Conservation Diversity*, 6, 372-380.

Martin, C.E., & Schmitt, K. (1989). Unusual water relations in the CAM atmospheric epiphyte *Tillandsia usneoides L.* (Bromeliaceae). *Botanical Gazette*, 150, 1-8.

Martinson, G.O., Werner, F.A., Scherber, C., Conrad, R., Corre, M.D., Flessa, H., Wolf, K., Klose, M., Gradstein, S.R., & Veldkamp, E. (2010). Methane emissions from tank bromeliads in neotropical forests. *Nature Geoscience,* 3, 766 -769.

Martorell, C., & Ezcurra, E. (2007). The narrow-leaf syndrome: A functional and evolutionary approach to the form of fog-harvesting rosette plants. *Oecologia,*  151(4), 561-567.

O Meara, G. F., Cutwa, M. M., & Evans, L. F. (2003). Bromeliad-inhabiting mosquitoes in south Florida: native and exotic plants differ in species composition. *Journal of Vector Ecology*, 28, 37-46

Oliveira, R. R. D. (2004). The importance of epiphytic bromeliads on the turnover of nutrients at the Atlantic Rain Forest. *Acta Botanica Brasilica*, 18(4), 793-799.

Ospina-Bautista, F., Estévez-Varón, J. V., Betancur, J., & Realpe-Rebolledo, E. (2004). Estructura y Composición de la comunidad de macro invertebrados acuáticos asociados a *Tillandsia turneri* Baker (Bromeliaceae) en un bosque Alto Andino Colombiano. Acta Zoológica Mexicana, 20(1), 153-166.

Ospina-Bautista, F., Estévez-Varón, J. V., Realpe, E., & Gast, F. (2008). Diversity of aquatic invertebrates associated to Bromeliaceae in the mountain cloud forest. Revista Colombiana de Entomología, 34(2), 224-229.

Richardson, B. A., Richardson, M. J., Scatena, F. N., & McDowell, W. H. (2000). Effects of nutrient availability and other elevational changes on bromeliad populations and their invertebrate communities in a humid tropical forest in Puerto Rico. *Journal of Tropical Ecology*, 16(2), 167-188.

Pierce, S., Winter, K., & Griffiths, H. (2002). The role of CAM in high rainfall cloud forests: an in situ comparison of photosynthetic pathways in Bromeliaceae. *Plant, Cell & Environment*, 25(9), 1181-1189.

Richardson, B. A., Rogers, C., & Richardson, M. J. (2000a). Nutrients, diversity, and community structure of two phytotelm systems in a lower montane forest, Puerto Rico. *Ecological Entomology*, 25(3), 348-356.

Richardson, B. A., Richardson, M. J., Scatena, F. N., & McDowell, W. H.  (2000b). Effects of Nutrient Availability and Other Elevational Changes on Bromeliad Populations and Their Invertebrate Communities in a Humid Tropical Forest in Puerto Rico. *Journal of Tropical Ecology*, 16(2), 167-188.

Santos, C. B. D., Leite, G. R., & Falqueto, A. (2011). Does native bromeliads represent important breeding sites for *Aedes aegypti* (L.) (Diptera: Culicidae) in urbanized areas? *Neotropical Entomology*, 40(2), 278-281.

Van Stan, J. T., & Pypker, T. G. (2015). A review and evaluation of forest canopy epiphyte roles in the partitioning and chemical alteration of precipitation. *Science of the Total Environment*, 536, 813-824.

Zotz, G. & Vera, T. (1999). How much water is in the tank? Model calculations for two epiphytic bromeliads. *Annals of Botany,* 83, 183-192.
