# Supplementary material for: Ecosystem services provided by bromeliad plants: A systematic review
Source: Ecol Evol. 2019 May 29;9(12):7360–72. doi: 10.1002/ece3.5296 (PMC6662323; doi:10.1002/ece3.5296)
Supplement: Supplementary file 5 [file ECE3-9-7360-s005.docx]

**APPENDIX 5.** References for cultural services provided by bromeliads.

| **Services** | **References** |
| --- | --- |
| Traditional knowledge | Agra *et al*., 2007; Albertasse *et al*., 2010; Bieski *et al*., 2012; Bieski *et al*., 2015; Bourdy *et al*., 2004; Coelho-Ferreira, 2009; De Almeida *et al*., 2011; De Feo & Soria, 2012; Juárez-Vásquez *et al*., 2013; Komlaga *et al*., 2015; Kujawska *et al*., 2012; Nunes *et al*., 2015; Oliveira *et al*., 2010; Samoisy & Mahomoodally, 2016; Sreekeesoon & Mahomoodally, 2014. |
| Aesthetic appreciation | Acebey *et al*., 2010; Hornung-Leoni, 2011; Irsyad *et al*., 2016; Mielke *et al*., 2009; Vanhoutte *et al*., 2016. |
| Cultural heritage | Baltazar Bernal *et al.,* 2014; Echeverry, 2011; Hornung-Leoni, 2011. |

**REFERENCES**

Acebey, A., Krömer, T., Maass, B. L., & Kessler, M. (2010). Ecoregional distribution of potentially useful species of Araceae and Bromeliaceae as non-timber forest products in Bolivia. *Biodiversity and Conservation*, 19(9), 2553-2564.

Agra, M. D. F., Baracho, G. S., Nurit, K., Basílio, I. J. L. D., & Coelho, V. P. M. (2007). Medicinal and poisonous diversity of the flora of “Cariri Paraibano”, Brazil.*Journal of Ethnopharmacology*, 111(2), 383-395.

Albertasse, P. D., Thomaz, L. D., & Andrade, M. A. (2010). Medicinal plants and their uses in Barra do Jucu community, Vila Velha Municipality, Espírito Santo State, Brazil. *Revista Brasileira de Plantas Medicinais*, 12(3), 250-260.

[Baltazar Bernal, O.](https://www.cabdirect.org/cabdirect/search/?q=au%3a%22Baltazar+Bernal%2c+O.%22),  [Zavala Ruiz, J.](https://www.cabdirect.org/cabdirect/search/?q=au%3a%22Zavala+Ruiz%2c+J.%22), [Solís Zanotelli, F. Y.](https://www.cabdirect.org/cabdirect/search/?q=au%3a%22Sol%c3%ads+Zanotelli%2c+F.+Y.%22),  [Pérez Sato, J. A.](https://www.cabdirect.org/cabdirect/search/?q=au%3a%22P%c3%a9rez+Sato%2c+J.+A.%22), & [Sánchez Eugenio, O.](https://www.cabdirect.org/cabdirect/search/?q=au%3a%22S%c3%a1nchez+Eugenio%2c+O.%22) (2014). Interpretive hiking of orchids and bromeliads in Tepexilotla, Chocamán, Veracruz. *Revista Mexicana de Ciencias Agrícolas*,  5 (9), 1687-1699.

Bieski, I. G. C., Rios Santos, F., de Oliveira, R. M., Espinosa, M. M., Macedo, M., Albuquerque, U. P., & de Oliveira Martins, D. T. (2012). Ethnopharmacology of medicinal plants of the pantanal region (Mato Grosso, Brazil). *Evidence-Based Complementary and Alternative Medicine*, Article ID 272749, 36 pages.

Bieski, I. G. C., Leonti, M., Arnason, J. T., Ferrier, J., Rapinski, M., Violante, I. M. P., Balogu, S.O., Pereira, J.F., Figueiredo, Rde.C., Lopes, C.R., de Silva, D.R., Pacini, A., Albuquerque, U.P., & Martins, D.T. (2015). Ethnobotanical study of medicinal plants by population of valley of Juruena region, legal Amazon, Mato Grosso, Brazil. *Journal of Ethnopharmacology*, 173, 383-423.

Bourdy, G., de Michel, L. C., & Roca-Coulthard, A. (2004). Pharmacopoeia in a shamanistic society: the Izoceno-Guaranı (Bolivian Chaco). *Journal of Ethnopharmacology*, 91(2), 189-208.

Coelho-Ferreira, M. (2009). Medicinal knowledge and plant utilization in an Amazonian coastal community of Marudá, Pará State (Brazil). *Journal of Ethnopharmacology*, 126(1), 159-175.

De Almeida, C. D. F. C., Rangel, B., Ramos, M. A., Silva, R. R. V., de Melo, J. G., Medeiros, M. F. T.,Sousa, T.A., Santos, A.L.,Cavalcanti, E.L., Da Nobrega, R.R., & de Albuquerque, U.P. (2011). Intracultural variation in the knowledge of medicinal plants in an urban-rural community in the Atlantic Forest from Northeastern Brazil. *Evidence-Based Complementary and Alternative Medicine*, 2012, Article ID 679373, 15 pages.

De Feo, V., & Soria, R. M. U. (2012). Medicinal plants and phytotherapy in traditional medicine of Paruro Province, Cusco department, Peru. *Pharmacol Online*, 1, 154-219.

Echeverri, J.A., & Román-Jitdutjaaño, O. (2011). Witoto ash salts frome Amazon. *Journal of Ethonopharmacology*, 138 (2), 492-502.

Hornung-Leoni, C. T. (2011). Avances sobre usos etnobotánicos de las Bromeliaceae en Latinoamérica. *Boletín Latinoamericano y del Caribe de Plantas Medicinales y Aromáticas*, 10(4), 297-314.

Irsyad, M., Pasek, A. D. & Indartono, Y. S. (2016). An investigation of green roof deployment in Bandung City, Indonesia. *Journal of Engineering and Applied Sciences*, 11(11), 2528 - 2534.

Juárez-Vázquez, M. C., Carranza-Álvarez, C., Alonso-Castro, A. J., González-Alcaraz, V. F., Bravo-Acevedo, E., Chamarro-Tinajero, F. J., & Solano, E. (2013). Ethnobotany of medicinal plants used in Xalpatlahuac, Guerrero, Mexico. *Journal of Ethnopharmacology*, 148(2), 521-527.

Komlaga, G., Agyare, C., Dickson, R. A., Mensah, M. L. K., Annan, K., Loiseau, P. M., & Champy, P. (2015). Medicinal plants and finished marketed herbal products used in the treatment of malaria in the Ashanti region, Ghana. *Journal of Ethnopharmacology*, 172, 333-346.

Kujawska, M., Zamudio, F., & Hilgert, N. I. (2012). Honey-based mixtures used in home medicine by nonindigenous population of Misiones, Argentina. *Evidence-Based Complementary and Alternative Medicine*, Article ID 579350, 15 pages

Mielke, E.C., Ribeiro do Valle, F.J., Poliquesi, C.B., & Cuquel, F.L. (2009). Ornamental potential of native vegetation from the “Campos Gerais, Brazil”. *Acta Horticulturae*. 813, 317- 320.

Nunes, A. T., Lucena, R. F. P., dos Santos, M. V. F., & Albuquerque, U. P. (2015). Local knowledge about fodder plants in the semi-arid region of Northeastern Brazil. *Journal of Ethnobiology and Ethnomedicine*, 11(1), 12.

Oliveira, F. C. S., Barros, R. F. M., & Moita Neto, J. M. (2010). Medicinal plants used in rural communities from Oeiras Municipality, in the semi-arid region of Piauí State (PI), Brazil. *Revista Brasileira de Plantas Medicinais*, 12(3), 282-301.

Samoisy, A. K., & Mahomoodally, F. (2016). Ethnopharmacological appraisal of culturally important medicinal plants and polyherbal formulas used against communicable diseases in Rodrigues Island. *Journal of Ethnopharmacology*, 194, 803-818.

Sreekeesoon, D. P., & Mahomoodally, M. F. (2014). Ethnopharmacological analysis of medicinal plants and animals used in the treatment and management of pain in Mauritius. *Journal of Ethnopharmacology*, 157, 181-200.

Vanhoutte, B., Ceusters, J., & De Proft, M. P. (2016). The ‘tubing’ phenomenon in commercial cultivation of *Guzmania*: morphology, physiology and anatomy. *Scientia Horticulturae*, 205, 112-118.
